# Mathematical and Live Meningococcal Models for Simple Sequence Repeat Dynamics – Coherent Predictions and Observations

**DOI:** 10.1371/journal.pone.0101637

**Published:** 2014-07-07

**Authors:** Kristian Alfsnes, Xavier Raynaud, Tone Tønjum, Ole Herman Ambur

**Affiliations:** 1 Department of Microbiology, University of Oslo, Oslo, Norway; 2 Department of Microbiology, Oslo University Hospital (Rikshospitalet), Oslo, Norway; 3 Department of Mathematics, University of Oslo, Oslo, Norway; 4 Department of Microbiology and Infection Control, Akershus University Hospital, Lørenskog, Norway; Institut Jacques Monod, France

## Abstract

Evolvability by means of simple sequence repeat (SSR) instability is a feature under the constant influence of opposing selective pressures to expand and compress the repeat tract and is mechanistically influenced by factors that affect genetic instability. In addition to direct selection for protein expression and structural integrity, other factors that influence tract length evolution were studied. The genetic instability of SSRs that switch the expression of antibiotic resistance ON and OFF was modelled mathematically and monitored in a panel of live meningococcal strains. The mathematical model showed that the SSR length of a theoretical locus in an evolving population may be shaped by direct selection of expression status (ON or OFF), tract length dependent (*α*) and tract length independent factors (*β*). According to the model an increase in *α* drives the evolution towards shorter tracts. An increase in *β* drives the evolution towards a normal distribution of tract lengths given that an upper and a lower limit are set. Insertion and deletion biases were shown to skew allelic distributions in both directions. The meningococcal SSR model was tested *in vivo* by monitoring the frequency of spectinomycin resistance OFF→ON switching in a designed locus. The instability of a comprehensive panel of the homopolymeric SSRs, constituted of a range of 5–13 guanine nucleotides, was monitored in wildtype and mismatch repair deficient backgrounds. Both the repeat length itself and mismatch repair deficiency were shown to influence the genetic instability of the homopolymeric tracts. A possible insertion bias was observed in tracts ≤G_10_. Finally, an inverse correlation between the number of tract-encoded amino acids and growth in the presence of ON-selection illustrated a limitation to SSR expansion in an essential gene associated with the designed model locus and the protein function mediating antibiotic resistance.

## Introduction

Genetic simple sequence repeats (SSRs) are inherently unstable [1], [2]. Long homopolymeric repeats in codon pair usage are avoided in all kingdoms of life due to their mutability [3]. Yet, many microorganisms utilize such repeats to generate variability in genes under selection for diversification [4], typically antigenic epitopes in bacterial pathogens and commensals that are exposed to adaptive immune systems. The opportunistic pathogen *Neisseria meningitidis* (Mc) and the obligate pathogen *N. gonorrhoeae* have small hyper-dynamic genomes with elevated spontaneous mutation rates compared to other bacterial species [5]–[7]. High mutation rates give rise to a multitude of genomic variants that may expand the fitness landscape, depending on the selective forces. The hyper-mutability is essential to Mc adaptability at the cost of genomic stability [8]. Two major genetic mechanisms have evolved to drive this rapid genomic hyper-variation, namely slipped-strand mispairing during replication of repeats and intrachromosomal recombination between silent and expression loci, resulting in phase variation (PV) and antigenic variation, respectively [9], [10]. Depending on the context, repeat-mediated instability may be under positive or negative selection causing either the establishment and maintenance or avoidance of repeats, respectively. Essential genes, of which expression is required for survival, tend not to harbour SSRs due to the inherent risk of causing alterations in the reading frame. Genetic variability can also be caused and amplified by defects in DNA repair (mutators), through variations in post-translational modifications of regulatory proteins and through horizontal gene transfer [11]–[15]. These sources of genetic variation may overlap and influence each other such as in the PV glycosylation gene network [16] and through the destabilizing effect transforming DNA has on poly guanine (polyG) tract stability [17]. In addition, transformation has been considered an important source for genetic variability [18].

Contingency loci, a collective term for genes subjected to PV, are generally involved in the structure or biosynthesis of surface proteins with simple sequence motifs, such as polynucleotide tracts, located in coding or promoter regions [19], [20]. In PV genes, the expression may switch ON/OFF status (reversible expression) by frameshift alterations or promoter efficacy depending on *cis* acting factors, the genetic composition of the repeat, or external *trans* acting factors [21]–[23]. The major *cis* acting factors are repeat number, repeat unit length, repeat sequence and purity, whereas DNA-polymerases, RNase H and mismatch repair (MMR) are *trans* acting factors shown to affect PV frequencies in both prokaryotic and eukaryotic model systems [21], [22], [24]–[28]. Polymeric tract length alterations in bacteria have been shown to be directionally biased depending on tract length and the nature of the tract [22], [29]. A recent study of a homopolymeric PV loci in *Campylobacter jejuni* revealed a shift from an insertion bias for shorter polyG tract lengths (8–9 nt) to a deletion bias for longer polyG tract lengths (10–11 nt) [30]. SSR instability offer plasticity in a stochastic manner, spontaneously or as a response to environmental change [31], such as the host immune response [32], [33], competition and bacteriophage infection [4], [34], cationic antimicrobial peptides (CAMPs) [35] or e.g. temperature and pH [36]. An accidental human passage of an Mc strain showed that more differences had occurred in PV genes during growth *in vivo* than *in vitro*, highlighting the influence of selection on Mc adaptation by means of PV [37]. The abundant Correia elements found in neisserial genomes have also been shown to provide a potential mechanism for PV by their unstable function as strong promoters [38]. PV may be considered a programmed event as the distribution of error-prone repeats is not random, making certain shifts in ON→OFF expression more likely to happen [32]. Based on comparative whole genome analyses in Mc and *N. gonorrhoeae*, more than 100 putative PV genes have been proposed and revised [39]–[43]. Homopolymeric tracts in naturally occurring PV loci in *Neisseria sp*. have been shown to vary considerably in length ranging from 7 nt to 19 nt [43], [44]. PV frequencies between 10^−4^ and 10^−5^ have been documented in Mc and these have been shown to change in both a tract length dependent manner and due to the influence of MMR [27], [45], [46]. A polyG tract length of nine nucleotides (G_9_), representing the ON-state of a haemoglobin binding protein (HmbR), was shown to be under positive selection in Mc disease isolates compared to carrier isolates [44]. In the same study, the ON-state (G_10_ and G_13_) of the genes encoding the haptoglobin-haemoglobin binding complex (HpuAB) was overrepresented compared to the OFF-state in both carrier and disease isolates. A recent study found reduced expression states of multiple PV loci during long-term Mc carriage, in part due to antibody-mediated selection [47]. These studies illustrate that selection for expression of individual loci may fluctuate between ON- and OFF-states and is thereby responsible for the evolution of PV. Through their contribution to epitope variation, PV genes enable Mc cells to evade host immune responses [32], [33], [48], providing a substantial challenge in making vaccines with persistent and broad protection [49].

Base-base mismatches appear spontaneously during replication either on the synthesis or the template strand causing insertions and deletions events (for review see [21]). Most of such errors are accurately repaired by post-replication MMR [50]. It is during these replication steps that the repeat tracts of PV genes switch between ON/OFF states, thus MMR limits or counteract PV [46]. The correlation between MMR and PV has been particularly well documented in Mc [27], [48] and defective MMR is a characteristic of Mc serogroup A disease suggesting that hyper-mutability may play a major role in virulence and infectivity [45]. Furthermore, Mc MMR deficiency has been found to enhance the escape from the bactericidal activity of a monoclonal antibody by PV of a relevant lipopolysaccharide gene (*lgtG*) [48]. Mc and many other bacterial entities of the β- and γ-Proteobacteria lack MutH in a methyl-independent MMR and the two main MMR components are therefore limited to MutS and MutL [15]. It was previously reported that Mc MMR components may be transiently titrated during uptake of DNA during Mc transformation, demonstrating that the PV frequency may increase during transformation and PV modulation by MMR may vary with the mismatch load [17].

## Materials and Methods

### Mathematical model of homopolymeric tract length evolution

#### Probabilistic model

We consider homopolymeric tracts, which can have lengths varying from 5 to 13 nucleotides. Given a homopolymeric tract, let us denote by *G(t)* its length, which is a function of time. The function *G(t)* takes value in the set {5,…,13}. For the sake of presentation, we introduce *G_i_ = i* and denote by *L* the set of all possible tract lengths (*L* = {5,…,13}). In this section, we focus on the probabilistic evolution of the tract length of a homopolymeric tract. In section 2 we introduce selection and model the effect on tract length evolution.

In an interval of time *Δt*, a homopolymeric tract of length *G_i_* may acquire a new nucleotide and thus increase its length by one. The probability for such an event is γ*_i_*Δ*t* where γ*_i_* is a given constant. It means that we have:
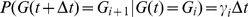
(1)


That is, the probability that a tract with a length *G_i_* at time *t* ends up with a length *G_i+1_* at time *t*+Δ*t* is equal to γ*_i_*Δ*t*. Similarly for nucleotide deletions we have:
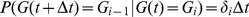
(2)where δ*_i_* is a given constant, that is, the probability that a tract of length *G_i_* at time *t* ends up with a length *G_i_*_−*1*_ at time *t*+Δ*t* is equal to δ*_i_*Δ*t*. For simplicity we assume that those probabilities are linear within the range (*L* = {5,…,13}) so that they can be written as:
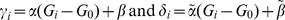
where *α*, *β*, 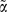 and 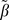 are constants that designate the ability to make mistakes by slippage. *α* and 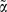 are here modelled to influence the probability of generating tract length alterations (γ and δ) in a tract length dependent manner (*cis* acting factor). *β* and 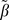 are constants that designate the baseline instability in a tract length independent manner (*trans* acting factor).

Let us now derive the evolution equations of the probabilities 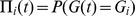


We have:
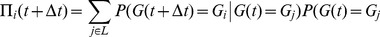
(3)


The probability that a tract length changes by one unit is given in (1) and (2). Furthermore, we assume that the event that a tract length changes by more than one unit occurs as a combination of successive single unit changes. Then, the probability of such an event is negligible with respect to Δ*t* when Δ*t* tends to zero and (3) can be rewritten as:
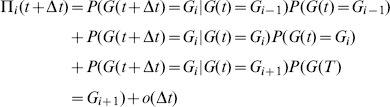
(4)


Here, *ο*(Δ*t*) denotes a function satisfying 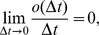
that is, a function that is negligible with respect to Δ*t*, when Δ*t* tends to zero. Since P is a probability, we have:
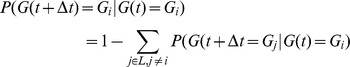
which can be rewritten as:
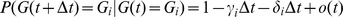
and equation (4) becomes:
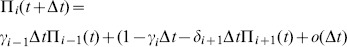
(5)


From equation (5), after dividing by Δ*t* and letting Δ*t* tend to zero, we obtain the following ordinary differential equation for ∏*_i_*,
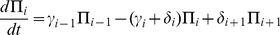
(6)


We assume that the insertion (*γ*) and deletion (*δ*) probabilities are equal, that is, *α* = 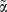 and *β* = 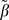. However, this may not necessarily be true *in vivo* and we also simulate two cases representing an insertion and a deletion bias. We then model the linear increase in tract instability (*γ* and *δ*) of hypothetical DNA repair deficient populations (e.g. MMR deficient), attempting to illustrate how DNA repair deficiency may influence the total instability relative to a wt population. The MMR deficient populations are assigned a higher initial *β* compared to the wt population. We then model three different scenarios: *i*) *α* in the MMR deficient population is identical to the wt population 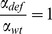, *ii*) *α* for the MMR deficient population is higher than in the wt population, and the *α* ratio between the MMR deficient population and the wt is smaller than the *β* ratio 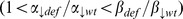, and finally *iii*) where the *α* ratio and the *β* ratio are equal in the two populations 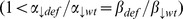.

#### The evolution of tract length distributions under the influence of selection

Here we tested the effect of selection (ON or OFF) on homopolymeric tract length evolution. We denote *μ_i_* the reproduction rate and *d_i_* the death rate of organisms with tract lengths *G_i_*. We know that organisms harbouring homopolymeric tract lengths that are multiples of 3 allows for genetic expression (ON), so that, in the presence of selection for expression, their death rate becomes much lower than for organisms with tract lengths that are not multiples of 3. We can model that by taking:
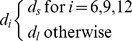
where *d_s_* is a *small* mortality rate and *d_l_* is a *large* mortality rate.

Let us now denote by *S_i_*(*t*) the number of homopolymeric tracts of length *i* for a given time *t*. We consider a system where resources are limited and introduce a carrying capacity *K*. Then, adding birth, death rate to the model equation (6), we obtain the following evolution equation for *S_i_*:
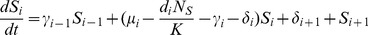
(7)Where 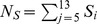
denotes the total population.

We make the simplifying assumptions that *μ_i_ = μ* does not depend on the DNA tract length, that the probabilities of nucleotide insertions and deletions are equal and that they are an increasing function of the length. Thus, the system can be rewritten as:
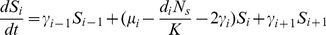
(8)


We solve Equation (8) and look at the distribution of organisms with respect to their length at equilibrium (that is after a long time). The parameters *α*, *β*, were manipulated while all other parameters were kept constant in order to investigate tract length evolution.

We model the scenario where there is selection against genetic expression (OFF), decreasing the death rate for tract lengths that are not multiples of 3, by taking:
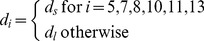



We use this to model the allelic distribution at steady-state equilibrium and to model an oscillating effect of ON/OFF selection. In addition, we also include scenarios of no selection (constant death rate *d_i_* for all tract lengths), tract length dependent death rate (*d_i_* increasing by tract length), and combination of tract length dependent death rate and ON or OFF selection (both *d_s_* and *d_l_* increase by tract length).

### Mapping the PV loci in Mc genomes

In order to obtain an overview of the distribution of homopolymeric tract lengths in Mc, known PV loci in three sequenced and annotated Mc genomes (MC58, Z2491 and FAM18) were compiled and their respective tracts compared (Table S1). In the few cases of conflicting data, the most recent references were used. Additional BLAST searches and alignments were conducted to confirm previously published data and to address PV loci in Mc strains lacking data.

### Bacterial strains, media and growth conditions

Mc and *Escherichia coli* strains used in this study are listed in Table 1; *N. meningitidis* serogroup A strain Z1099 was employed as in a previous publication [51] and grown on commercially available and CE marked 5% human blood agar plates or GC agar plates at 37°C with 5% CO_2_, while *E. coli* was grown on Luria-Bertani (LB) agar at 37°C. For selection, Mc was grown in the presence of 100 µg ml^−1^ kanamycin (Kan), 3 µg ml^−1^ erythromycin or 50 µg ml^−1^ spectinomycin (Spc), and for *E. coli* 50 µg ml^−1^ Kan or 50 µg ml^−1^ ampicillin when appropriate.

**Table 1:** Plasmids, primers and strains.

Plasmids:	Description	Reference
pARR2107	*spOFF* and *erm*-resistance gene flanked by Mc *hpuB*	[1]
pBluescript SKII+ (pBSKII+)	Cloning vector, Amp^R^	Stratagene
pUP6	Cloning vector pHSS6 modified with DUS and reverse DUS, Kan^R^	[2]
pUD8	pARR2107 with inserted 12-mer DUS downstream of *spOFF*, with G_8_ tract	This study
pUD5	Same as above, but with G_5_ tract	This study
pUD7	Same as above, but with G_7_ tract	This study
pUD10	Same as above, but with G_10_ tract	This study
pUD11	Same as above, but with G_11_ tract	This study
pUD13	Same as above, but with G_13_ tract	This study
pO9	pARR2107 with inserted 12-mer DUS downstream of *spOFF*, with 9 nt non-repeat tract in ON-state	This study
pO12	Same as above, but with 12 nt non-repeat tract in ON-state	This study
pOHA*mutL::aph*	pBSK II+ harboring *mutL* from MC58 with inserted kanamycin resistance marker *aph*	This study
**Strains:**	**Description**	**Reference**
*N. meningitdis* strain Z1099	Serogroup A, isolated in the Philippines in 1968	Dominique A. Caugant
*E. coli* strain ER2566	Electrocompetent cells prepared following standard protocol	New England Biolabs
**Primers:**	**Description**	**Sequence**
7160SAF-Tn5-*aph*-for	Construction of pOHA*mutL::aph*	GCGGATCCTAGACTGGGCGGTTTTATGG
7161SAF-Tn5-*aph*-rev	Construction of pOHA*mutL::aph*	GCTCTAGATCATTTCGAACCCCAGAGTC
7162OHA101	Construction of pOHA*mutL::aph*	CGCTCGAGGGCGGATTCAGTCTTGGTAA
7163OHA106	Construction of pOHA*mutL::aph*	GCGGATCCTTACTGTCCGCGCAAGAACA
7164 OHA105	Construction of pOHA*mutL::aph*	GCTCTAGAGCCGAAAGTGCTAGAATACG
7165OHA104	Construction of pOHA*mutL::aph*	GCGAGCTCCGTTTTGAGCATAGCGTTGA
KA24	pUD7	GTACGGCTCTGCAGTGGAT**GGGGGGG**CTGAAGCCACACAG
KA25	pUD7	CTTCAG**CCCCCCC**ATCCACTGCAGAGCCGTACAAATGTAC
KA26	Sequencing primer	GTGATCGCCGAAGTATCGAC
KA27	Sequencing primer	AGTTCGCGCTTAGCTGGATA
KA28	pUD5	GTACGGCTCTGCAGTGGAT**GGGGG**CTGAAGCCACACAG
KA29	pUD5	CTTCAG**CCCCC**ATCCACTGCAGAGCCGTACAAATGTAC
KA30	pUD10	GTACGGCTCTGCAGTGGAT**GGGGGGGGGG**CTGAAGCCACACAG
KA31	pUD10	CTTCAG**CCCCCCCCCC**ATCCACTGCAGAGCCGTACAAATGTAC
KA32	pUD11	GTACGGCTCTGCAGTGGAT**GGGGGGGGGGG**CTGAAGCCACACAG
KA33	pUD11	CTTCAG**CCCCCCCCCCC**ATCCACTGCAGAGCCGTACAAATGTAC
KA91	pUD13	GTACGGCTCTGCAGTGGAT**GGGGGGGGGGGGG**CTGAAGCCACACAG
KA92	pUD13	CTTCAG**CCCCCCCCCCCCC**ATCCACTGCAGAGCCGTACAAATGTAC
KA111	pO9	GTACGGCTCTGCAGTGGAT**GGCGGCGGCC**TGAAGCCACACAG
KA112	pO9	CTTCAG**GCCGCCGCC**ATCCACTGCAGAGCCGTACAAATGTAC
JEE7	pO12	**GGCGGCGGCGGC**CTGAAGCCACACAGTGATATTGATTTG
JEE8	pO12	**GCCGCCGCCGCC**ATCCACTGCGGAGCCGT

1. Alexander HL, Richardson AR, Stojiljkovic I (2004) Natural transformation and phase variation modulation in Neisseria meningitidis. Mol Microbiol 52: 771–783.

2. Wolfgang M, Van Putten JPM, Hayes SF, Koomey M (1999) The comP locus of Neisseria gonorrhoeae encodes a type IV prepilin that is dispensable for pilus biogenesis but essential for natural transformation. Mol Microbiol 31: 1345–1357.

### Preparation of PV constructs with different repeat lengths

Plasmids employed are listed in Table 1. The panel of pUD plasmids was transformed into Mc serogroup A strain Z1099 to allow the *aadA* reporter gene with varying polyG tract lengths to integrate into the *hpuB* locus in the chromosome as previously described [17], [51]. The *aadA* gene encodes 3″-adenylyl-transferase conferring spectinomycin (Spc) resistance. Spc is an aminocyclitol, closely related to the aminoglycosides, which is bacteriostatic by interfering with the mRNA-ribosome interaction. In this live Mc model, AadA expression (ON) is achieved by the spontaneous nucleotide insertion or deletion of the polyG tract inserted into the coding region of the *aadA* gene encoding the fortieth amino acid and onwards [17]. The *aadA* reporter model is artificial in that it is not a PV gene in nature and is made by substituting a non-homopolymeric Gly-codon pair with consecutive polyG Gly-codons. Out-of-frame polyG tracts G_5_, G_7_, G_10_, G_11_ and G_13_ were made by site-directed mutagenesis of the G_8_ tract designed by Alexander and co-workers [17] following standard protocols from Stratagene [52], using *Pfu* Turbo polymerase and primers listed in Table 1. In order to switch into frame, each construct required a single nt insertion or deletion as listed in Table 2. Two non-switching controls, O_9_ and O_12_, in which the polyG tracts were replaced by non-homopolymeric codons (i.e. not GGG) transcribing the same amino acid (Gly), were constructed (Table 2). Plasmids were transformed into *E. coli* strain ER2566 and repeat lengths were confirmed by DNA sequencing using an ABI BigDye Terminator v.3.1 DNA sequencing kit (Applied Biosystems) and primers listed in Table 1. Transformation of the plasmids into Mc strain Z1099 with an intact MMR was performed essentially as described previously [53]. Single colonies were cloned and the individual polyG repeat lengths were confirmed by PCR using Phusion High-Fidelity Polymerase (Finnzymes) to minimize stutter product formation [54] and subsequent DNA sequencing was undertaken as described above.

**Table 2:** Sequence of the polyG tract constructs.

Constructs	PolyG tract region	Required OFF→ON mutation	# of AA as ON*
**G_5_**	GTG GAT **GGG GG**- CTG AAG CCA	+1 nt (to G_6_)	2×Gly
**G_7_**	GTG GAT **GGG GGG G**– CTG AAG CCA	−1 nt (to G_6_)	2×Gly
**G_8_**	GTG GAT **GGG GGG GG**- CTG AAG CCA	+1 nt (to G_9_)	3×Gly
**G_10_**	GTG GAT **GGG GGG GGG G**– CTG AAG CCA	−1 nt (to G_9_)	3×Gly
**G_11_**	GTG GAT **GGG GGG GGG GG**- CTG AAG CCA	+1 nt (to G_12_)	4×Gly
**G_13_**	GTG GAT **GGG GGG GGG GGG G**– CTG AAG CCA	−1 nt (to G_12_)	4×Gly
**O_9_**	GTG GAT **GGC GGC GGC** CTG AAG CCA	In-frame	3×Gly
**O_12_**	GTG GAT **GGC GGT GGA GGG** CTG AAG CCA	In-frame	4×Gly

*This lists the number of amino acids (AA) only in the tract as seen bold in the table, Gly = Glycine.

### Preparation of MMR null mutants

To study the effect of a defect in the MMR pathway on PV frequency, Mc null mutants were constructed using a *mutL* knock-out plasmid pOHA*mutL::aph* (Table 3). The pOHA*mutL::aph* containing the neisserial MMR gene *mutL* interrupted by a selective marker (*aph*) encoding Kan resistance was made using primers listed in Table 1, following standard protocols and procedures. Transformations of the pOHA*mutL:aph* into Mc with polyG tract constructs were conducted as described above.

**Table 3:** OFF→ON Switching frequencies.

Initial tract length*	Median (range) (wt)	Step-wise comparison (wt)**	Median (range) (MMR)	Step-wise comparison (MMR)**	Comparison wt & MMR deficient**	Comparison repeat & non-repeat tract**
**G_5_** (G_6_)	7.7×10^−12^ (5.1×10^−12^–2.7×10^−11^)		1.7×10^−09^ (6.8×10^−12^–2.1×10^−05^)		−52.9, *ns*	N/A
		−43.3, *ns*		−67.2, *p*≤0.05		
**G_7_** (G_6_)	6.5×10^−10^ (1.4×10^−11^–1.5×10^−07^)		1.9×10^−07^ (1.6×10^−10^–2.1×10^−05^)		−76.7, *p*≤0.05	N/A
		−50.5, *ns*		−44.1, *ns*		
**G_8_** (G_9_)	2.4×10^−08^ (1.3×10^−09^–2.1×10^−06^)		9.3×10^−06^ (1.7×10^−08^–1.0×10^−03^)		−70.3, *p*≤0.05	−147.2, *p*≤0.001 (**O9**)
		−20.2, *ns*		38.4, *ns*		
**G_10_** (G_9_)	1.2×10^−07^ (3.0×10^−10^–1.1×10^−05^)		5.2×10^−07^ (3.2×10^−10^–1.4×10^−05^)		−11.7, *ns*	−96.7, *p*≤0.05 (**O9**)
		57.7, *ns*		77.1, *ns*		
**G_11_** (G_12_)	1.2×10^−09^ (1.2×10^−11^–5.3×10^−07^)		1.2×10^−09^ (4.6×10^−11^–1.1×10^−07^)		7.7, *ns*	−111.9, *p*≤0.05 (**O12**)
		−0.5, *ns*		−26.0, *ns*		
**G_13_** (G_12_)	4.5×10^−09^ (1.4×10^−11^–2.8×10^−07^)		1.1×10^−08^ (1.0×10^−10^–4.0×10^−07^)		−17.9, *ns*	−111.5, *p*≤0.05 (**O12**)
		-				
**O_9_** (in-frame)†	0.6 (0.5–1.1)		N/A		N/A	-
		22.3, *ns*		N/A		
**O_12_** (in-frame)†	2.2×10^−04^ (2.4×10^−07^–5.6×10^−04^)		N/A		N/A	-

*Initial tract length and closest ON configuration in (), see Table 2.

**Kruskal-Wallis with post Dunn's multiple comparison tests, difference in rank sum.

† Median survival frequencies are shown for the two non-repeat controls (O_9_ and O_12_).

No significant difference denoted by “*ns*”.

Non appropriate comparison denoted by “-”.

Not available denoted by “N/A”.

### PV assay

Mc wildtype (wt) and *mutL* mutants were grown on commercially available and CE marked 5% human blood agar plates. Single colonies were resuspended in 200 µL 1× phosphate buffered saline (PBS) in multiple well plates (96 wells – round-bottom, Sarstedt, US). For the constructs with very short or long polyG tract (G_5_, G_7_ and G_13_), pooled suspensions of up to 10 single colonies were used to allow the appearance of sufficient numbers of colonies on selective plates. Serial dilutions for the assessment of spontaneous mutations and total number of Mc colony forming units (CFUs) were prepared using 20 µL stocks and 180 µL 1× PBS, from which 100 µL of the appropriate dilution was then spread on selective (Spc) and non-selective blood plates, respectively. The polyG constructs were designed and confirmed to be initially out-of-frame (OFF), while rescued growth on selective media enabled a direct measure for the tendency of each tract to switch into reading frame (ON). The two non-repeat controls, O_9_ and O_12_, were measured as survival frequency as these already expressed AadA and Spc resistance. In this assay, PV occurs during non-selective growth by slipped-strand mispairing in the replication of a polyG tract inside the model gene, *aadA*. A subset of the initial Spc sensitive clone thereby becomes Spc resistant through nt insertions or deletions of the polyG tract, as defined in Table 2, allowing AadA expression. Switching frequencies are as such a measure of the proportion of a single colony that expresses AadA prior to selection. Notably, the proportion of a colony that expresses AadA is dependent on both the rate of switching, but also the number of cell divisions that follow switches, which produce clonal descendants. The introduction of Spc selection represents a bottleneck that is expected to allow the exclusive establishment of colonies from CFUs or parts thereof that have switched to an in-frame configuration and their non-switched descendants. The switching frequencies were studied for the panel of different repeat lengths in two backgrounds, wt and MMR deficient. Due to substantial variation in switching frequencies in replicate experiments and non-parametrically distributed data, trends of instability were monitored using median switching frequencies as in previous studies [27], [46], [55] and found to be the most acceptable approach in predicting mutation rates [56]. Statistical analyses of the non-parametric switching frequencies were performed using Kruskal-Wallis one-way analysis of variance with post Dunn's multiple comparison tests.

### Colony size measurement

The range of colony sizes for each Spc resistant polyG construct and the non-repeat control was investigated in order to determine a suspected influence by the number of tract encoded amino acids in AadA on growth in the presence of Spc. Full resolution images of randomly chosen antibiotic resistant colonies following the PV assay were obtained after 48 hours growth using a Leica DC300 digital camera fitted onto a Leica MZ16A magnifier. Images were analyzed using the ImageJ software setting the threshold at the maximum extent of the colonies, measuring the area in mm^2^. Care was taken to avoid the effect of nutrient depletion or other density-dependent growth inhibition by studying isolated single colonies located several colony diameters away from other colonies. The parametric colony size distribution and correlation with tract length were calculated using the Student's t-test.

## Results

### Mathematical model of SSR dynamics and the influence on tract length distributions

**Figure 1:**
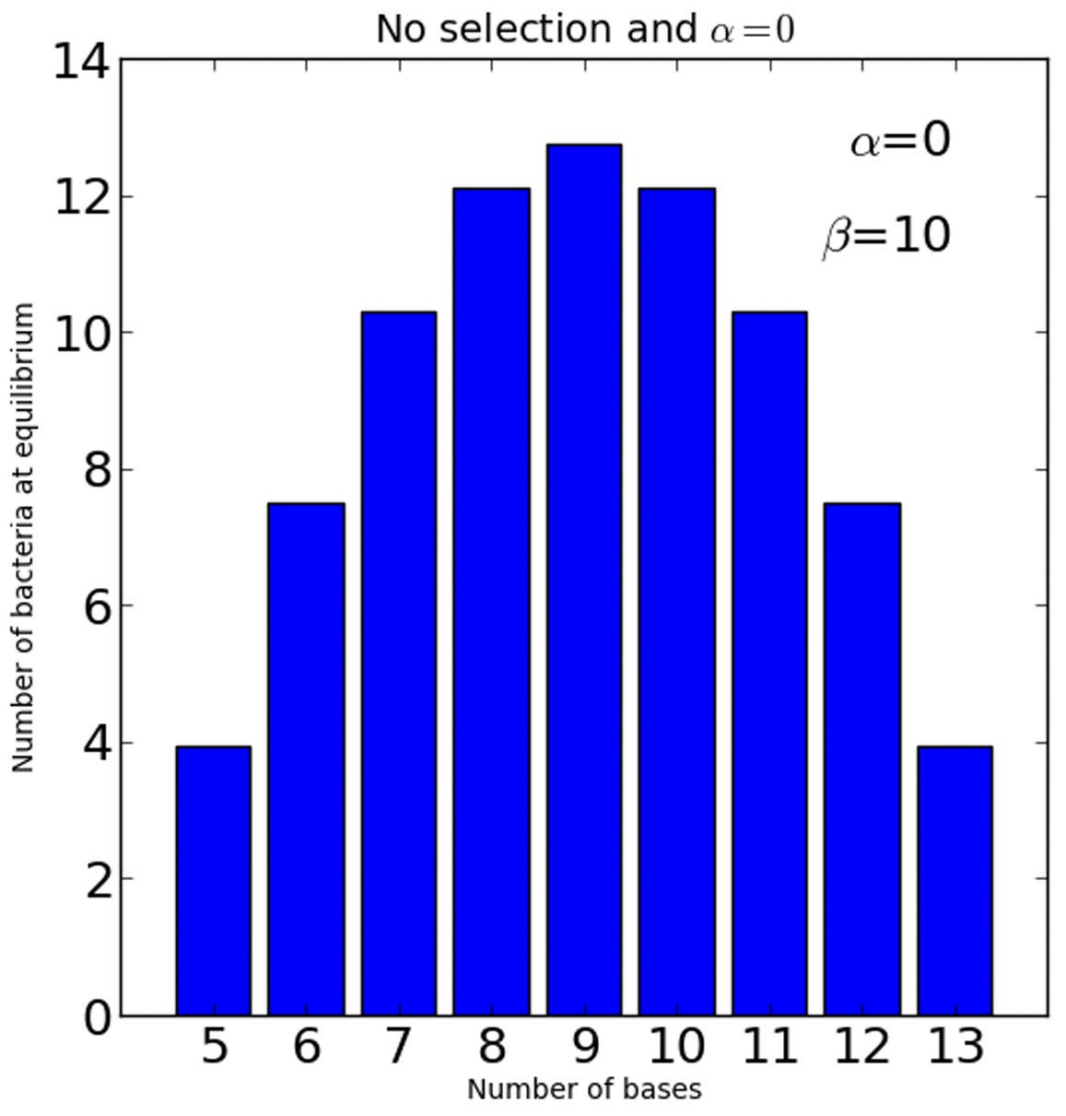
Mathematical model illustrating random walk. Allelic steady-state distribution is shown of a modelled bacterial population without selection and no contribution of the *cis* acting factor *α*. The figure illustrate the isolated effect of the *trans* acting factor *β* on the distribution of the tract lengths, an effect caused by increased random walk within a defined range of tract lengths. Where *K* = 100, *μ* = 5, and *d_both_* = 5.

A mathematical model was developed to study the evolutionary dynamics of a homopolymeric SSR using a set of simple parameters as described in the materials and methods. First, the probabilistic evolution of homopolymeric tract length was modelled considering a *cis* acting factor, tract length dependent instability (*α*), and a *trans* acting factor, tract length independent instability (*β*). *β* effectively describes a baseline instability that is equal for all tract lengths. Figure 1 shows the influence of *β* on tract length evolution in the absence of *α*, without penalizing selection (see below) and when the range of possible tracts is limited upwards (13 nt) and downwards (5 nt). When the tract range is limited, the evolution of tract lengths takes a normal distribution.

Secondly, we modelled a linear increase in total instability (*γ* and *δ*) of hypothetical DNA repair deficient populations compared to a wt population (Figure S1). In this model, the values of *α* and *β* for the DNA repair deficient populations were factors of *α* and *β* from the wt population. By altering the *α* ratios in the MMR deficient populations we observed: *i*) and *ii*) a relative decreasing influence on instability by MMR deficiency as tracts become longer, *iii*) the level of instability of the MMR deficient population remains twice that of the wt population, regardless of tract length (Figure S1).

**Figure 2:**
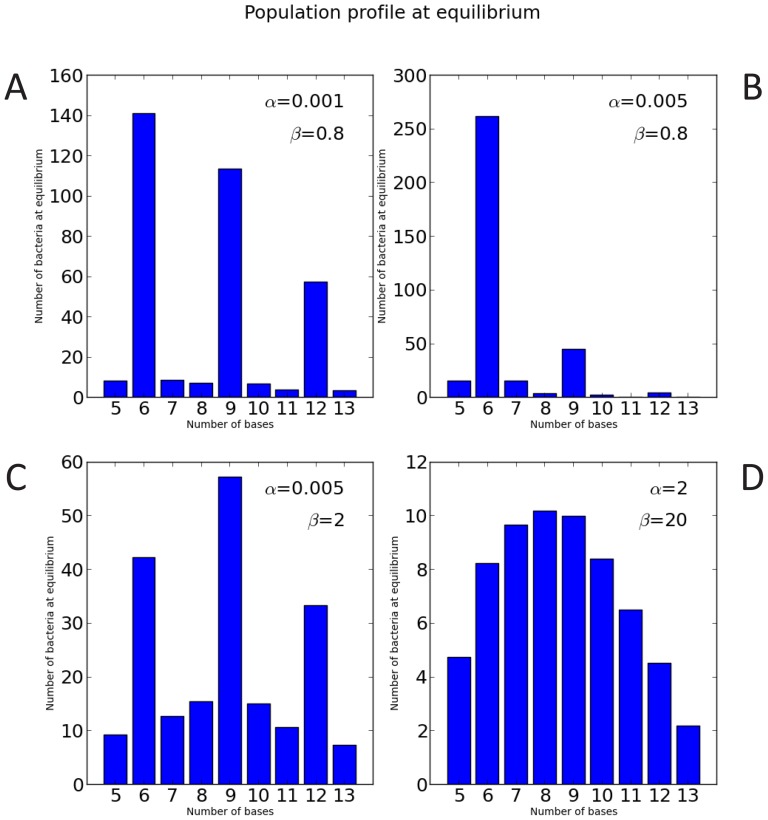
Mathematical modelling of the evolution of homopolymeric tract length. Allelic steady-state distributions are shown following different values of tract length dependent instability (*α*) and tract length independent instability (*β*) (panel A–D). The influence of selection was taken into account by implementing penalizing selection on tracts that were not multiples of 3, corresponding to the out-of-frame tract length selection seen in the PV assay. Where *K* = 100, *μ* = 5, *d_s_* = 1 and *d_l_* = 5.

Thirdly, the influence of penalizing selection was modelled by implementing higher mortality rates for tracts that were not multiples of 3, analogous to the in-frame tract length that allows AadA expression (ON) in the PV assay. Figure 2 A–D shows the steady-state allelic distribution of homopolymeric tracts in model populations that experience penalizing selection and differential levels of genetic instability (tract length dependent *α* and independent *β*). Panel A shows the steady-state distribution of alleles in a population that experiences relatively low levels of instability from both *cis* (*α* = 1.0×10^−03^) and *trans* (*β* = 0.8) factors. The alleles most frequently expressed in the steady-state population in Panel A are G_6_, G_9_ and G_12_, representing the in-frame (ON) tract lengths. By increasing the *cis* factor five-fold (*α* = 5.0×10^−03^), the allelic distribution shifts towards the shorter in-frame tract (G_6_) as shown in panel B. By comparing panel B to panel C, it is shown that by increasing the *trans* factor 2.5-fold (*β* = 2), the allelic distribution shifts towards a normal distribution within the defined range (5–13 nt). Comparing panel C and panel D, the *cis* factor is increased 400-fold (*α* = 2) and the *trans* factor is increased tenfold (*β* = 20), keeping the distribution biased towards shorter tracts and in a near normal distribution.

We observed that an insertion bias (*γ*>*δ*) drives the population of tract lengths at equilibrium towards longer tracts (Figure S2, panels C and D) which peak at G_9_ compared to a deletion bias (*γ*<*δ*) which peak at shorter tract lengths (Figure S3).

It can be assumed that tract length evolution in nature fluctuate between periods of no selection, selection for expression and selection for no expression. Figure S4 shows the death rate imposed by these three different scenarios and Figure S4 case1–3 the steady-state outcomes. With a constant death rate and no selection (Figure S4 case1), the steady-state of the allelic distribution remains equal to or similar to normal distribution (panels A–C). When *α* is increased the distribution is skewed towards shorter tracts (panel D). In the scenario with OFF selection (Figure S4 case 3), the allelic distributions mirrors that with ON selection as shown in case 2 (Figure S4 case 2) and Figure 2; again an increase in *α* skews the population towards shorter tracts (panels A and B), while an increase in *β* forces the distribution to approach normal distribution and reduces the total number of bacteria in the population at equilibrium (panels C and D). An oscillating effect between ON and OFF selection is shown in Video S1 (and the corresponding time average in Figure S5). In light of the PV assay and colony size measurements demonstrating reduced fitness/higher mortality rates for longer in-frame tracts (G_9_ and G_12_) compared to shorter tract (G_6_) (see below), we introduced a tract length dependent death rate (Figure S4 case 4). Under this scenario, the steady-state allelic distribution is skewed towards shorter tracts (panels A and B). An increase in *α* and particularly *β* are required to skew the distribution towards longer tract lengths (panel C and D), again with a dramatic reduction of the total number of bacteria in the population. Different scenarios for selection (ON or OFF) were combined with the tract length dependent death rate to show their influence on tract length evolution (Figure S4 case 5 and Figure S4 case 6). Similar to the tract length dependent death rate (Figure S4 case 4), the scenarios for selection (ON or OFF) combined with a tract length dependent death rate show that the steady-state allelic distribution is skewed towards shorter tracts when *α* and *β* are low (panels A and B in Figure S4 case 5 and Figure S4 case 6). For the scenario with tract length dependent death rate and ON selection (Figure S4 case 5), the increase in *β* reduces the total number of bacteria at equilibrium (panels B and C). A further increase in both *α* and *β* force the population towards normal distribution, causing further reduction in the total number of bacteria at equilibrium (panel D). In the final scenario, with tract length dependent death rate and OFF selection (Figure S4 case 6), an increase in *β* when *α* is relatively low allows the allelic distribution to drift towards longer tracts (panel C). Further increases in *α* and *β* force the population towards normal distribution, again effectively reducing the total number of bacteria at equilibrium (panel D).

### The distribution of PV homopolymeric tracts in three Mc strains

**Figure 3:**
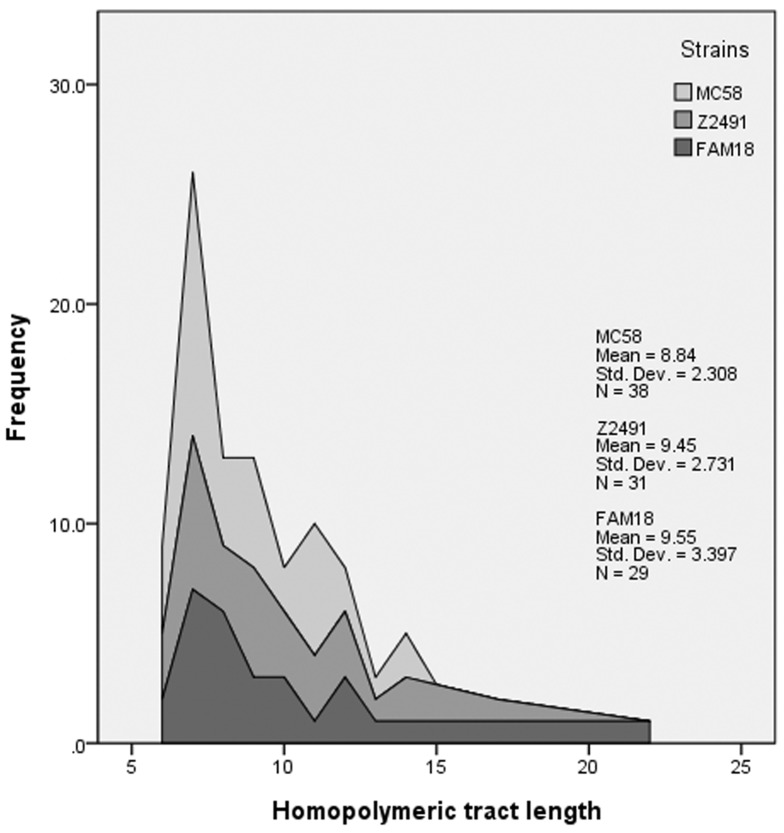
Distribution of homopolymeric tract lengths in Mc strains. A comparison of the homopolymeric tract lengths of putative PV genes identified in three different Mc strains (see references in Table S1).

The distribution of naturally occurring PV loci in Mc was reviewed here in order to understand the biologically relevant tracts and tract length range as studied in a live model. In Mc, out of 79 putative PV genes investigated, 50 were represented with homopolymeric tracts, 21 of which were polyC, while 18 were polyG, 8 were polyA and only 1 polyT, relative to the transcriptional direction of the individual gene (Table S1). Two additional genes, *mtfB* and *dnaX*, had different homopolymeric motifs in different strains and are not included here. Out of the 79 putative PV genes, 68 of the genes were represented with intragenic repeat tracts (Table S1). The distribution of the homopolymeric tract lengths of putative PV loci in three different Mc genomes were found to be skewed towards shorter tracts, with the majority of loci constituted by 7 nt repeat tracts (Figure 3). Notably, many PV genes remain unverified and may be the consequence of other drivers than fluctuating selection for expression such as GC content harmonization. Also, the individual tract lengths listed in Table S1 are snapshots in time of an individual genome and are expected to vary when subject to selection in live populations. Average tract lengths in all three strains were found to be close to 9 nt (8.84, 9.45 and 9.55 for strains MC58, Z2491 and FAM18, respectively) (Figure 3 and Table S1). The average length of homopolymeric tracts consisting of C/G was not found to be statistically different from tracts consisting of A/T (*p* = 0.096).

### Switching frequencies are influenced by tract length and MMR deficiency

In a live Mc model, a PV assay was employed to investigate switching frequencies (OFF→ON) of Spc resistance in a panel of polyG tracts accommodated inside the model gene encoding AadA. From the start codon, the polyG tracts were constructed at 118 nt and onwards inside the 795 nt *aadA* gene corresponding to amino acids 40 and onwards as initially described by Alexander *et al.* [17]. Rescued growth on selective media containing Spc enabled a measure for the tendency of each polyG tract to switch OFF→ON expression of Spc resistance. The range of polyG tracts accommodated in *aadA*, 5 to 13 nt in length, as listed in Table 2, was monitored in the PV assay. Additionally, two non-repeat control strains (O_9_ and O_12_), both in the ON-configuration, were included (Table 2).

**Figure 4:**
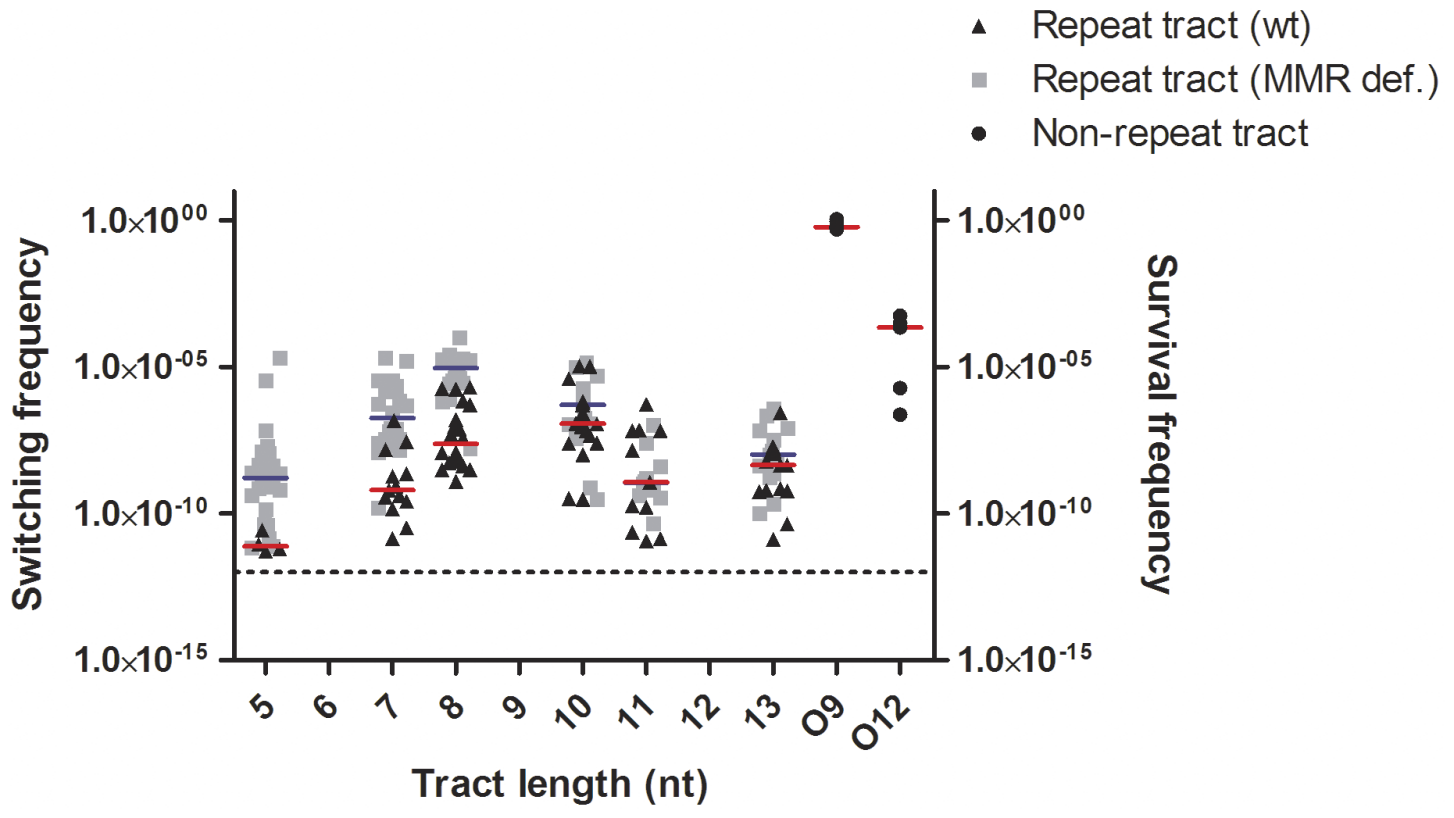
Switching frequencies of a range of polyG tracts *in vivo* in Mc wt and MMR deficient backgrounds. Switching frequencies (left y-axis) are shown for each tract length G_5_ to G_13_ in wt (black pyramids), the MMR deficient background (grey squares), and the survival frequencies (right y-axis) for the non-repeat controls (black circles). Median value indicated for each tract length for wt (red) and MMR deficient (blue) background. Dotted line indicates the spontaneous mutation frequency for Spc resistance (1.0×10^−12^).

In the wt background, the median switching frequency in the strain harbouring the shortest polyG tract (G_5_) was found to just exceed that of the spontaneous mutation frequency to Spc resistance (7.7×10^−12^ vs 1.0×10^−12^), which is known to appear in Mc due to mutations in 16S rRNA [57] (Figure 4 and Table 3). The G_7_ strain switched with two orders of magnitude higher median frequency to ON (6.5×10^−10^) than G_5_ (7.7×10^−12^). Sequencing of Spc resistant colonies in the PV assay confirmed that both G_5_ and G_7_ switched to G_6_, the nearest ON configuration that required a single nucleotide insertion/deletion, respectively. The median switching frequency observed with the G_8_-strain (2.4×10^−08^) was two orders of magnitude higher than the frequency of the G_7_ strain and hence four orders higher than the G_5_. The switching frequency of G_10_ (1.2×10^−07^) was found to be only one order of magnitude higher than G_8_, significantly different from G_7_ (*p*<0.05) and the highest recorded median frequency in the wt background. Sequencing of the polyG tract in Spc-resistant colonies confirmed that both G_8_ and G_10_ switched to G_9_, the nearest ON configuration that required a single nucleotide insertion/deletion, respectively. Both G_11_ (1.2×10^−09^) and G_13_ (4.5×10^−09^) switched to the nearest ON configuration G_12_ with median frequencies two orders of magnitude lower than the frequency of G_10_. The median switching frequencies in the wt background were found to be higher, but not statistically significant, for tract lengths requiring a deletion (G_7_, G_10_ and G_13_) compared to tract lengths requiring an insertion (G_5_, G_8_ and G_11_) (Table 3). Two ON strains with non-repeat tracts were included in the PV assay as controls. The O_9_ strain displayed a median switching frequency (ON→OFF) close to 1 (0.6) (i.e. 100% survival) and in comparison the O_12_ strain displayed a median survival frequency (2.2×10^−04^) four orders of magnitude lower than O_9_. All median survival frequencies in the control strains differed significantly from the switching frequencies in the relevant polyG strains as outlined in Table 3.

In order to investigate the association between PV and MMR, MutL null mutants were made for each individual Mc strain harbouring different polyG tract lengths in *aadA*, and these were monitored in the same manner as the strains in the wt background. In the MMR background, a similar stepwise increase in switching frequencies was observed in the G_5_ to G_8_ range as for that observed in the wt background (Figure 4 and Table 3). The G_7_ MMR strain switched with two orders of magnitude higher median switching frequency (1.9×10^−07^) than G_5_ (1.7×10^−09^) and thereby differed significantly from G_5_ (*p*≤0.05). Furthermore, the median switching frequency observed with the G_8_-strain (9.3×10^−06^) was the highest recorded in the assay and nearly two orders of magnitude higher than the frequency of the G_7_ strain (1.9×10^−07^) and hence nearly four orders higher than that of the G_5_ (*p*<0.001). The median switching frequency of the G_10_ (5.2×10^−07^) was somewhat lower compared to G_8_ (9.3×10^−06^), and the difference is not significant. The median switching frequencies of the G_11_ (1.2×10^−09^) and G_13_ (1.1×10^−08^) strains were further reduced by more than two orders of magnitude compared to the G_8_ strain and the differences were significant (*p*<0.001 and *p*<0.05, respectively), displaying a similar pattern to that of the wt. Taken together, MutL-derived MMR deficiency was shown to increase the median switching frequency relative to wt by nearly three orders of magnitude in individual comparisons in the G_5_-G_8_ range. No difference was observed in comparing switching frequencies in MMR and wt strains with polyG tracts in the G_10_–G_13_ range.

### Colony size correlates inversely with polyG tract length after growth on selective media

**Figure 5:**
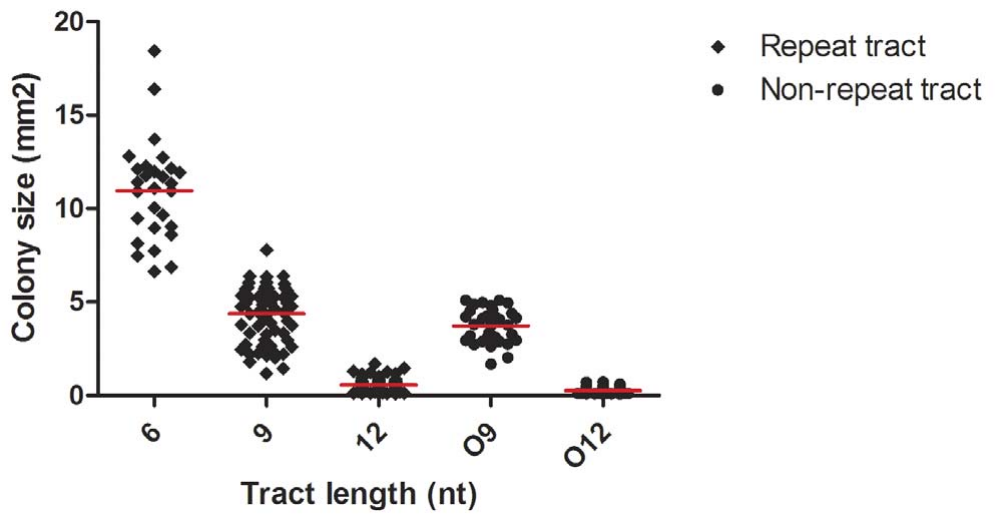
An inverse relationship between Mc colony size and homopolymeric tract length. Colony sizes (area) of CFUs following the PV assay are shown for the in-frame tract length of the PV constructs (black diamonds), and the non-repeat controls (black circles). Mean value for each in-frame tract length indicated (red).

The results from the PV assay described above did not display an association between the longest tract lengths (>10 nt) and the highest frequencies of switching that was expected. This observation warranted further investigation. It was noticed during the PV assay that strains with different polyG tract lengths not only switched to resistance with different frequencies, but also that these strains displayed varying colony sizes only when grown on selective media (Figure S6). Strains with the shorter tract lengths seemed to yield larger colonies than strains with longer tracts. Therefore, the colony size (area) of re-plated ON strains in both backgrounds were measured after 48 h growth on selective media. The data was grouped by ON tract lengths (i.e. G_5_/G_7_ = G_6_, G_8_/G_10_ = G_9_ and G_11_/G_13_ = G_12_) (Figure 5 and Table 4). No statistical differences in colony size were found within each group or between backgrounds (wt vs. MMR deficient), nor between strains grown in the absence of Spc selection. However, highly significant differences were found (*p*<0.001) between groups G_6_ vs. G_9_ and between G_9_ vs. G_12_ (and G_6_ vs. G_12_) where G_6_ were the largest and G_12_ the smallest colonies. G_9_ colonies were on average smaller than half the average G_6_ colony and the average G_12_ colony was yet smaller than half the average G_9_ colony.

**Table 4:** Colony size (mm^2^).

In-frame tract length*	Mean (95% CI)	Step-wise comparison**	Comparison repeat & non-repeat tract**
**G_6_**	10.9 (9.9–12.0)		N/A
		*t* = 15.2, *df* = 91, *p*≤0.0001	
**G_9_**	4.4 (4.0–4.7)		*t* = 2.3, *df* = 95, *p*≤0.05 (O9)
		*t* = 23.4, *df* = 151, *p*≤0.0001	
**G_12_**	0.6 (0.5–0.6)		*t* = 6.2, *df* = 138, *p*≤0.0001 (O12)
**O_9_**	3.7 (3.4–4.1)		-
		*t* = 26.4, *df* = 82, *p*≤0.0001	
**O_12_**	0.3 (0.2–0.3)		-

*In-frame ON tract length, see Table 2.

**Unpaired Student's t-test, two-tailed p-value.

Non appropriate comparison denoted by “-”.

Not available denoted by “N/A”.

## Discussion

Mathematical modelling of the dynamic allelic distribution of homopolymeric tracts in a population predicted that tract length dependent, *cis* (*α*) and tract length independent *trans* (*β*) acting factors of stability influence tract length evolution differently. *β* designates the baseline instability that corresponds to the probability for making a transition to a different length. Transitions happen randomly (unless a bias is applied) and the *β* function causes random walk. An increase in *β* allows the allelic distribution to drift towards longer tracts where *α* is increased. In the absence of *β* the allelic distribution may not drift away from the shorter tracts where the effect of *α* is lower. The random walk effect of *β* is illustrated in Figure 1, where there is no penalizing selection and *α* = 0, illustrating how the allelic population is forced towards a normal distribution by the set tract length range. *In vivo* this range may be defined by yet other factors such as biases in proofreading (insertion/deletion) and protein constraints. A high tract length dependent *α* favoured shorter tracts over the course of evolution (Figure 2, panels A and B). In the extreme example with high *α* and *β* (Figure 2 panel D), not even the penalizing large death rate (*d_l_*) that applies to tract lengths that are other than multiples of 3, is sufficiently powerful to prevent near complete fluidity between allelic forms. The increased *β* forces a normal distribution of the allelic forms by increasing the probability for making a transition to a different length in the manner of random walk. The penalizing selection (*d_l_*) also becomes more efficient when tracts are rapidly changing away from tract lengths with lower death rate (*d_s_*). These increasingly forced transitions to a different tract length have in turn a dramatic effect on fitness of the population as shown by the relative few surviving bacteria of each allelic form at equilibrium (Figure 2, panel D).

Alterations of the tract length occur due to polymerase slippage during replication [58]–[60]. Given that an ideal mutation rate exists at a particular locus at a moment in time, an error-prone polymerase may be able to provide that instability using shorter tracts than a high fidelity polymerase. However, overexpression of the single error-prone polymerase (DinB) in Mc does not cause a global mutator phenotype such as in *E. coli* [46]. Also, tract lengths may not exclusively evolve from their ability to fine-tune an ideal mutation rate but may be determined by molecular drivers [30].

Hypothetical MMR deficient populations were modelled with higher *β* values, and *α* values as functions of *α* in the wt population (Figure S1). This approximation may not fully represent the *in vivo* state since MMR deficiency also could be represented with *β* as a factor of *α*. In the PV assay however, MMR deficiency was found to cause 200–400-fold increase in instability of the G_5_, G_7_ and G_8_ tracts. Therefore, within the defined range of tracts, a linear model may prove to be a useful approximation. The model allowed us to visualize a range of different contributions of MMR deficiency in a tract length dependent manner. The different scenarios modelled included: *i*) parallel increase in instability in wt and MMR populations where the contribution of MMR relative to the total instability decreased, *ii*) increased relative contribution of MMR and *iii*) a constant contribution of MMR deficiency to the total instability for all tract lengths. The relative reduction in the contribution of the modelled MMR deficiency may be similar to the decreasing effect of MMR deficiency with increasing tract length that was observed in the PV assay (see below for G_8_/G_10_ comparison). DNA repair deficient strains known as mutator strains generate a broad spectrum of variants at great cost as most mutations are deleterious (in our model those generating G_5_, G_7_, G_8_, G_10_, G_11_, G_13_ when ON-selection applies) and fewer are advantageous (G_6_, G_9_ and G_12_). Despite the costs, mutator strains have been shown to be successful in several models and organisms [61], [62].

Previous studies of the instability of long (>30 nt) repeat tract lengths in bacteria have revealed a deletion bias [22], [29], [63], [64], while for eukaryotic cells a possible insertion bias has been proposed [24], [29]. In *C. jejuni*, Bayliss and co-workers showed that biases in the mutational pattern changed from predominantly insertions for G_8_ and G_9_ to predominantly deletions for G_10_ and G_11_ [30]. This shift may potentially be related to how indels predominantly are repaired by polymerase proofreading in short tracts to MMR in longer tracts as discussed in [30]. A possible insertion bias for G_8_ and G_10_ was observed in the PV assay (see below). Adjusting the mathematical model with insertion/deletion bias revealed how the steady-state population tends towards longer and shorter tracts for an insertion bias and a deletion bias, respectively (Figures S2 and S3, see panels C and D).

The mathematical model makes the assumption that the reproduction rate is independent of the tract length, whereas the death rate is dependent of the tract length, shown in Figure 2, with small death rates (*d_s_*) for tract lengths in multiples of 3 (G_6_, G_9_ and G_12_), and large death rates (*d_l_*) for all other tract lengths (G_5_, G_7_, G_8_ etc.). Findings from the model PV assay used here and colony size measurements indicate reduced fitness/survival for long in-frame tracts compared to short tracts (see below), as such we modeled different scenarios of death rate (*d*); no selection, ON/OFF selection, linear increasing death rate, and combination of linear increasing death rate and ON/OFF selection (Figure S4). A scenario with fluctuating ON/OFF selection (Figure S4, cases 1 and 2; Video S1) may effectively describe the natural evolution of PV loci in Mc, and the model PV assay employed in this study (see below). The oscillating ON/OFF selection (Video S1) shows the dynamic shifts in the allelic distribution of tract lengths that on average approach a normal distribution with median tract length at 9 (Figure S5). When comparing the allelic distribution of the time average to the corresponding allelic distributions of a single ON or OFF selection scenario with the same *α* and *β* values (panel B in Figure S4 case 2 and case 3), it becomes evident that modeled populations under oscillating selection can maintain longer tract lengths. This may explain how longer repeat tracts are maintained in bacteria in nature i.e dependent on oscillating selection.

The review of the existing literature addressing neisserial PV events revealed a total of 87 putative PV genes in the three selected Mc genomes, all three with average homopolymeric tract length of ∼9 nt (Figure 3). It has been shown by Markov chain analysis of the MC58 genome that C/G-tracts longer than 6 nt, and particularly 8 nt, were found in considerably higher excess than expected in this organism [39]. Contrary to a previous report [39], this study did not reveal any statistical difference between homopolymeric C/G and A/T tract lengths. The distribution of the homopolymeric tracts identified in this study exhibits a bias towards C/G repeats (39∶9) and intragenic location (41∶9) as shown in Table S1, whereas genomic analysis of hypothetical homopolymeric tracts in the coding region has revealed an underrepresentation of C/G repeats with less observed tracts than expected from random distribution [2]. The overrepresentation of homopolymeric tract lengths around 9 nt may indicate a suitable range for balancing variation and conservation, where short tracts will be too stable and long tracts will be too unstable [2], [65], [66]. A previous study has shown how evolutionary pressures may mould such PV loci, showing that the position of the repeat in pathogenic bacteria is suppressed in the middle and enriched near the termini of the genes [2]. Notably, many of the PV loci listed in Table S1 are not verified as PV genes and the presence of SSRs may be the consequence of e.g. GC content harmonization. Also, the individual tract lengths listed in Table S1 are snapshots in time of an individual genome and are expected to vary when subject to selection in live populations. Further characterization of individual putative PV loci and genome comparisons are thus warranted to better understand SSR evolution.

This study investigated the extent to which the length of a polyG tract and MMR influences tract instability by monitoring shifting expression of an antibiotic resistance reporter, AadA, conferring Spc resistance. Mc PV frequencies have been found to differ depending on the PV monitoring system used [17]. Alexander and co-workers found that strains devoid of haemoglobin utilization (Δ*hmbR/*Δ*hpuB*) continued growth for ∼3 divisions when cultivated on iron-limited media causing an overestimation of *hmbR* OFF→ON switching compared to *aadA* Spc^S^→Spc^R^ switching [17]. A model PV assay of Spc^S^→Spc^R^ was therefore employed in the live model presented here. The range of switching frequencies observed in the model PV assay were found to be lower compared to previously published Mc PV frequencies (10^−4^ to 10^−5^) [27], [45], [46] and to PV frequencies in other bacteria (10^−2^ to 10^−4^) [22], [30]. This may be due to strain differences. Previous publications have noted many fold differences in switching frequency of constructed PV loci between different Mc strains (10^−2^ to 10^−6^) [20], [27]. The observed PV frequencies for Z1099, a Mc serogroup A strain, fall within the frequency range previously observed for a Mc serogroup A strain with overexpressed MutS (∼10^−7^) [17]. Z1099 does therefore exhibit switching frequencies in the lower range of previously published frequencies, and the comparative status of DNA repair in this strain remains to be investigated at the molecular level.

The results from the PV assay indicate an expected stepwise increase in switching frequencies with increased tract length (from G_5_ to G_8_), most probably due to the tract length dependent instability (a *cis* acting factor). The stepwise log-linear increase in PV for the repeat tract lengths G_5_ to G_8_ was comparable to previous studies using the same basic construct [45], and with homopolymeric repeat tracts in yeast [24]. The median frequency observed for G_10_ deviates from the log-linearity with an increase only one order of magnitude higher than that of G_8_ in the wt background. This increase is less than expected since G_10_ is two nucleotides longer than G_8_ and hence expected to be considerably more unstable due to tract length dependent instability. Comparatively there is an increase in median frequency of two orders of magnitude when the tract is increased with a single nucleotide from G_7_ to G_8_. These observations may indicate an insertion bias in tracts G_7_, G_8_ and G_10_. An insertion bias would increase the difference between G_7_ and G_8_ and decrease the difference between G_8_ and G_10_ since both G_7_ and G_10_ require a deletion to reach the closest ON-state whereas G_8_ requires an insertion. The possible insertion bias finds further support when studying the frequencies in the MMR deficient background as discussed below. The deviation from log-linearity in switching frequencies observed for repeat tract lengths longer than G_10_ are likely due to altered protein function. This phenomenon may be due to a reduction in fitness from decreased antibiotic resistance effectiveness of the AadA protein. Such an effect may in turn be ascribed to the differences in the number of transcribed amino acids from the polyG tract inside *aadA*. When Alexander and co-workers initially inserted the polyG tract in *aadA*, they replaced a non-homopolymeric pair of Gly codons (6 nt) with a homopolymeric and incomplete triplet of Gly codons (8 nt) [17]. *aadA* is not a PV gene in nature but a model locus applied here. The different ON repeat tract lengths in the coding region of *aadA* encode two (G_6_), three (G_9_ and O_9_) or four (G_12_ and O_12_) Gly-amino acids. The efficiency of the protein may be influenced by the number of amino acids encoded by the polyG region, with a drastically reduced efficiency when the tract encodes four amino acids, as such reducing the viability of the bacterial cells despite being in an ON-configuration. Structural predictions of homologous AadA in *Salmonella enterica* indicate that the PV tract resides in a β-loop in between and in close proximity to conserved regions [67]. Increasing the tract length and thereby the number of amino acids may therefore cause subtle alterations in the three-dimensional protein configuration that reduce, but notably do not abolish, adenylyl transferease activity. The survival frequencies monitored in the non-repeat controls supported this hypothesis, since the survival frequency of the 4 × Gly (O_12_) differed from a frequency of 1 and that of the 3×Gly (O_9_). Further support for this hypothesis was obtained by monitoring the colony size as discussed below.

The PV assay confirmed a well-established association between MMR and SSR instability [21], . The MutL-derived MMR deficiency was shown to increase switching frequencies 200–400-fold relative to wt within the G_5_-G_8_ range. With a *hmbR* PV assay, Alexander and co-workers have previously shown a 51–59 fold increase in switching frequencies of a G_8_ tract in a MMR deficient serogroup A Mc strain (IR4048) [20]. MMR contribution is considerably more pronounced (200–400-fold) in our observations using a different serogroup A strain (Z1099) and a different PV assay. Some of this difference may possibly be ascribed to the PV models in that the *hmbR* PV assay is less stringent than the Spc assay. However, differences in MMR efficacy in separate strains of variable genetic composition and context cannot be excluded. It is therefore interesting that a high incidence of MMR mutator strains in epidemiological isolates has been reported [45]. The strain used here (Z1099) may not accurately represent the whole spectrum of pathogenic Mc strains, but allude to the great variation in MMR influence on SSR instability.

In yeast, functional MMR has been shown to reduce insertion events in repeat tract lengths causing a deletion bias [59], as well as strongly affecting the fidelity of long repeat tract lengths [24]. Comparison of the PV rates in this study (Table 3) does not indicate that a deletion bias is alleviated in the MMR deficient background. On the contrary, a potential insertion bias, like in the wt background, is indicated in comparing G_8_ and G_10_ in the MMR deficient background (Figure 4). The median switching frequency of the shorter G_8_ requiring an insertion was near 20-fold higher than the longer G_10_ requiring a deletion. As for the wt background, we would expect the G_10_ frequency to greatly exceed that of G_8_ due to an increase in tract length dependent instability. An insertion bias for G_8_ (and G_9_) has also been documented in *C. jejuni* [30]. Furthermore, the difference in G_10_ median switching frequency of the wt and MMR deficient strains are considerably smaller (and not statistically significant) compared to the respective statistically significant differences for G_7_ and G_8_. Also it is notable that the frequencies of G_7_ and G_10_ (both requiring a deletion) in the MMR deficient background are very similar. These observations seem to suggest that, irrespective of a potential insertion bias, the influence of MMR deficiency on tract instability becomes less pronounced as tracts get longer as modelled in Figure S1. This notion is further supported by the lack of difference between backgrounds in G_11_ and G_13_ strains, although their respective frequencies are confounded by impaired growth.

When comparing the colony sizes of strains with different ON-tract lengths when grown on selective media (Spc), a highly significant negative correlation was documented between ON-tract length and colony size (Table 4 and Figure 5). A similar highly significant tendency was detected also in the non-repeat tract control strains (O_9_ and O_12_). The tendency for reduced colony size with increased tract length, could potentially be alleviated by reducing the selective pressure mediated by Spc, since colony sizes were equal in the absence of selection. Apart from the tract length, the strains were identical and the colony phenotype therefore seems exclusively associated with the drug resistance mechanism, inactivation of Spc by adenylylation, which is a characteristic of AadA. The original non-manipulated AadA has 2×Gly, and the manipulated 2×Gly strains monitored here give rise to the largest colonies, indicating the highest relative fitness of the panel. The fitness reduction in the 3×Gly and 4×Gly strains may be the most important factor to influence the PV assay in the longer tract range, overwhelming the monitored other *cis* (length) and *trans* acting (MMR) factors.

## Conclusions

A linear mathematical model of SSR dynamics allowed the study of how individual factors influenced evolution, alone and in concert with others. Values of the tract length dependent factor *α* were found to inversely correlate to tract length evolution illustrating the interplay between fidelity of DNA replication and SSR evolution. The baseline instability factor *β*, analogous to DNA repair deficiency, was shown a very powerful determinant of tract length evolution that, if exaggerated, had the potential to greatly reduce fitness of the population by forcing the generation of deleterious mutations. Theoretical scenarios were outlined describing how the relative contribution of MMR to the instability of individual tract lengths could differ. Some of these show how the relative contribution of MMR to tract instability decreases as the tract length dependent instability grows in accordance with observations of the *in vivo* model. MMR was shown to increase homopolymeric G tract instability 200–400-fold in strain Z1099, considerably higher than reported in other Mc strains. The mathematical model also illustrated how fluctuating selection for expression was required for the evolution of longer tracts and captured thereby the essence and dynamic rationale behind PV evolution. Molecular drivers of tract length evolution was modelled as insertion and deletion biases and found to drive the allelic distribution towards longer or shorter tracts, respectively. The experimental data suggested that tracts ≤G_10_ experienced an insertion bias; the first documentation of such phenomenon in Mc. Finally, this study highlights the notable influence of protein function on the potential for evolvability by means of PV and the *de novo* evolution of intragenic SSRs. PV loci may seem subject to a balancing act between tract elongation and tract contraction defining a tract length space within the borders of complete fluidity and inflexibility. The improved understanding of the dynamics of SSR and PV may shed light on how individual PV loci and thereby antigenic epitopes evolve, which is relevant for population dynamics, host-pathogen interactions and ultimately for vaccine development.

## Supporting Information

Figure S1 **Mathematical modelling of the relative contribution of a hypothetical MMR deficiency to tract instability.** The increase in instability (*γ* and *δ*) with increasing tract length is shown for a hypothetical wt population (blue), a MMR deficient population with *α* values equal to that of the wt population (green), a MMR deficient population with relatively higher *α* values to the wt deficient population (red) and a MMR deficient population where the *α* values are equal to the relative difference between *β* in the MMR deficient and the wt populations (turquoise).
(TIF)

Figure S2 **Mathematical modelling of the evolution of homopolymeric tract length with an insertion bias.** Allelic steady-state distributions as in Figure 2, with an insertion bias (*δ* = 0.8*γ*). The different panels A-D illustrate the allelic steady-state distribution of tract lengths following different values of tract length dependent instability (*α*) and tract length independent instability (*β*). Where *K* = 100, *μ* = 5, *d_s_* = 1 and *d_l_* = 5.
(TIF)

Figure S3 **Mathematical modelling of the evolution of homopolymeric tract length with a deletion bias.** Allelic steady-state distributions as in Figure 2, with a deletion bias (*γ* = 0.8*δ*). The different panels A-D illustrate the allelic steady-state distribution of tract lengths following different values of tract length dependent instability (*α*) and tract length independent instability (*β*). Where *K* = 100, *μ* = 5, *d_s_* = 1 and *d_l_* = 5.
(TIF)

Figure S4 **Mathematical modelling of different scenarios of selection.** Allelic steady-state distributions shown for different values of death rate/mortality *d_s_* and *d_l_* as depicted in the first slide: without selection (case 1), selection ON (case 2) and OFF (case 3), tract length dependent death rate (case 4), and combinations of ON/OFF selection and tract length dependent death rate (case 5 and 6). Panels A–D in each case 1–6 illustrate the allelic steady-state distributions of tract lengths following comparable values of tract length dependent instability (*α*) and tract length independent instability (*β*). Where *K* = 100 and *μ* = 5.
(ZIP)

Figure S5 **Time average of the mathematical modelled population with oscillating ON/OFF selection shown in Video S1.** Allelic time average distribution of the mathematical modelled population with oscillating ON/OFF selection (as shown in Video S1). In the modelled population *K* = 100, *μ* = 5, *d_s_* = 1 and *d_l_* = 5, where *d_s_* and *d_l_* oscillates for tract lengths that are multiple of three (6, 9, 12) and not multiple of three (5, 7, 8, 10, 11 & 13).
(TIF)

Figure S6 **Colonies after 48 hours of growth on Blood Spc50 plates for the panel of in-frame tract lengths in wt and non-repeat controls.** Depicting a selection of colonies representing the in-frame tracts G_6_ (for G_5_/G_7_), G9 (for G_8_/G_10_) and G_12_ (for G_11_/G_13_) and the non-repeat repeat tracts O_9_ (9 nt) and O_12_ (12 nt), the white bars represent 10 mm. Tract length constructs as described in Table 1 and 2.
(TIF)

Table S1 **List of putative phase variable loci in three Mc strains.**
(DOC)

Video S1 **Mathematical modelled oscillating ON/OFF selection.** The video illustrates the temporal shifts in allelic distribution when a mathematical modelled population is subjected to oscillating ON and OFF selection (non-oscillating ON and OFF selection shown in Figure S4, case 2 and 3). The population does not reach steady-state equilibrium as long as it is under oscillating penalizing selection pressure, a time average of the oscillating allelic distributions is shown in Figure S5. In the modelled population, *K* = 100, *μ* = 5, *d_s_* = 1 and *d_l_* = 5, where *d_s_* and *d_l_* oscillates for tract lengths that are multiple of three (6, 9, 12) and not multiple of three (5, 7, 8, 10, 11, 13).
(MP4)
