# Supplementary figures and images for: Mathematical and Live Meningococcal Models for Simple Sequence Repeat Dynamics – Coherent Predictions and Observations
Source: PLoS One. 2014 Jul 7;9(7):e101637. doi: 10.1371/journal.pone.0101637 (PMC4085013; doi:10.1371/journal.pone.0101637)

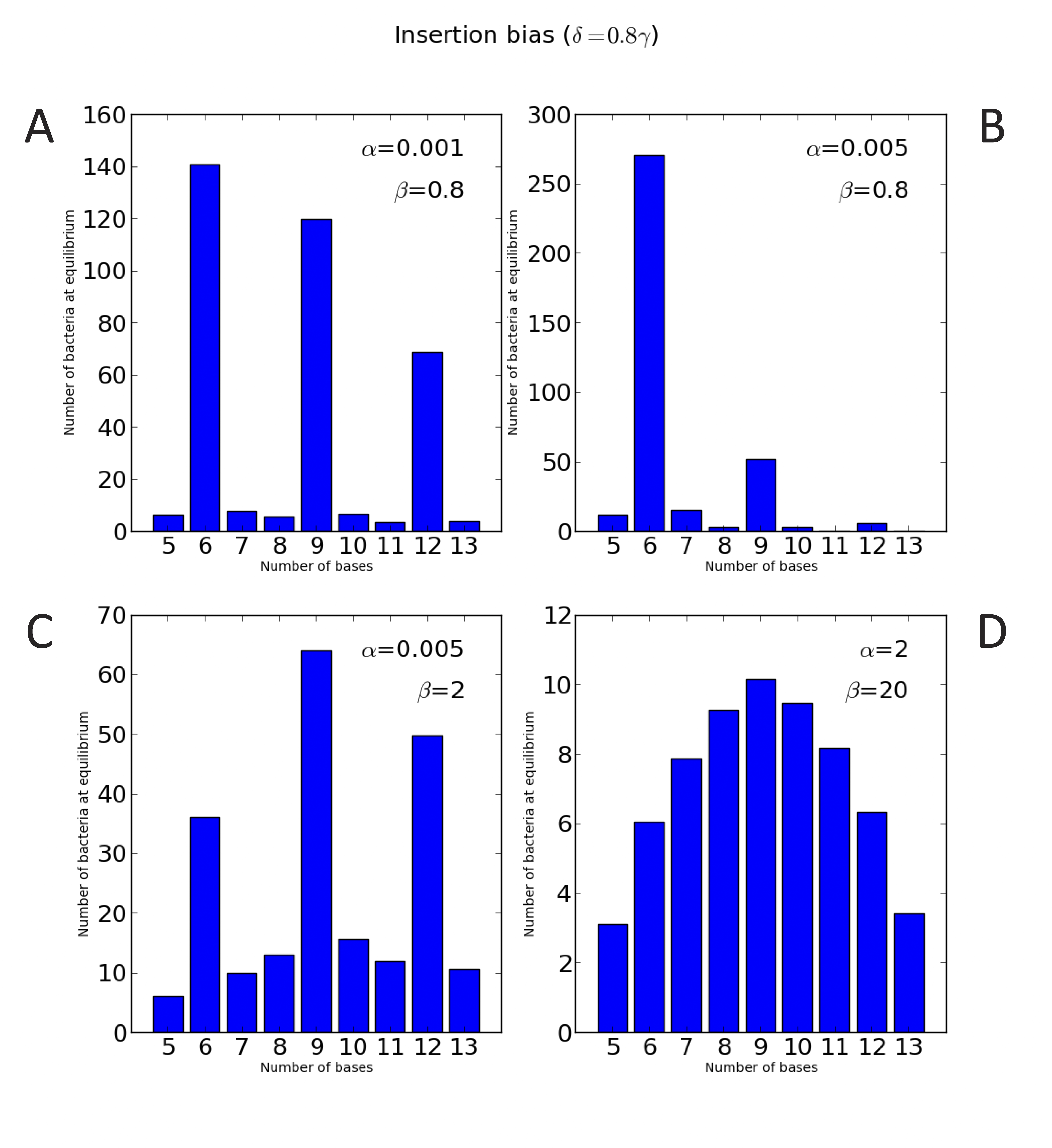

Supplement: Figure S2 — Mathematical modelling of the evolution of homopolymeric tract length with an insertion bias. Allelic steady-state distributions as in Figure 2, with an insertion bias (δ = 0.8γ). The different panels A-D illustrate the allelic steady-state distribution of tract lengths following different values of tract length dependent instability (α) and tract length independent instability (β). Where K = 100, μ = 5, ds = 1 and dl = 5. (TIF) [file pone.0101637.s002.tif]

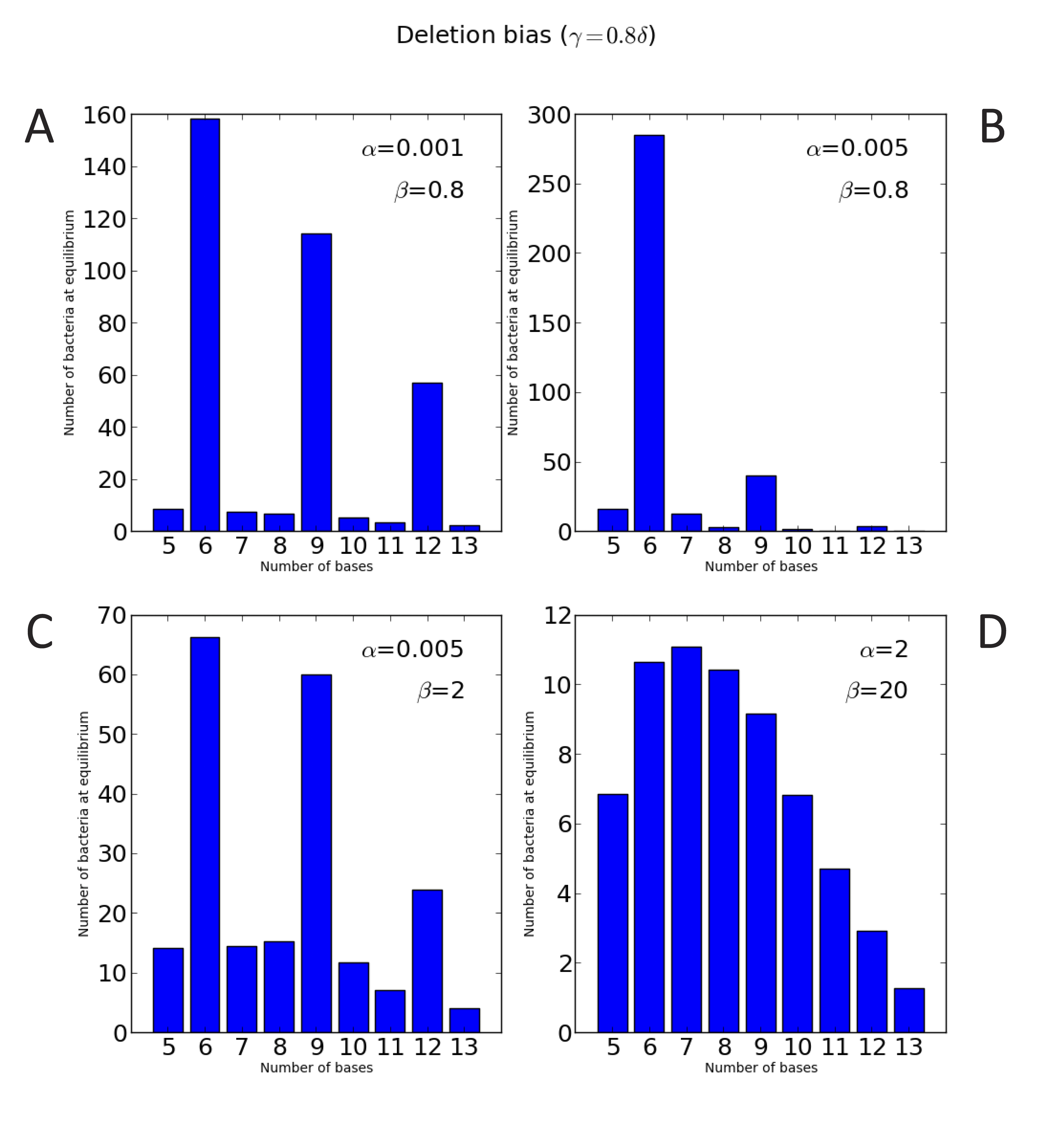

Supplement: Figure S3 — Mathematical modelling of the evolution of homopolymeric tract length with a deletion bias. Allelic steady-state distributions as in Figure 2, with a deletion bias (γ = 0.8δ). The different panels A-D illustrate the allelic steady-state distribution of tract lengths following different values of tract length dependent instability (α) and tract length independent instability (β). Where K = 100, μ = 5, ds = 1 and dl = 5. (TIF) [file pone.0101637.s003.tif]

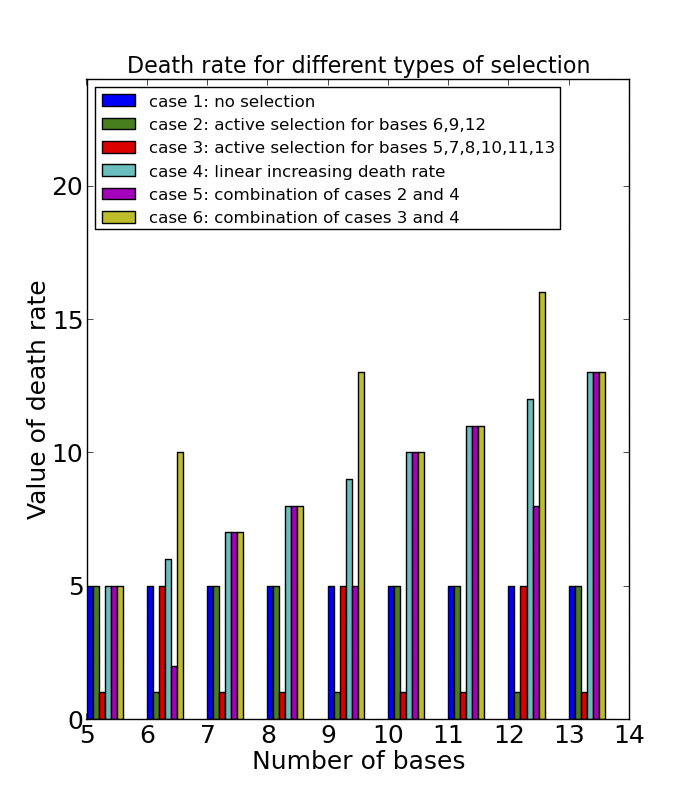

Supplement: Figure S4 — Mathematical modelling of different scenarios of selection. Allelic steady-state distributions shown for different values of death rate/mortality ds and dl as depicted in the first slide: without selection (case 1), selection ON (case 2) and OFF (case 3), tract length dependent death rate (case 4), and combinations of ON/OFF selection and tract length dependent death rate (case 5 and 6). Panels A–D in each case 1–6 illustrate the allelic steady-state distributions of tract lengths following comparable values of tract length dependent instability (α) and tract length independent instability (β). Where K = 100 and μ = 5. (ZIP) [file pone.0101637.s004.zip › FigS4.tif]

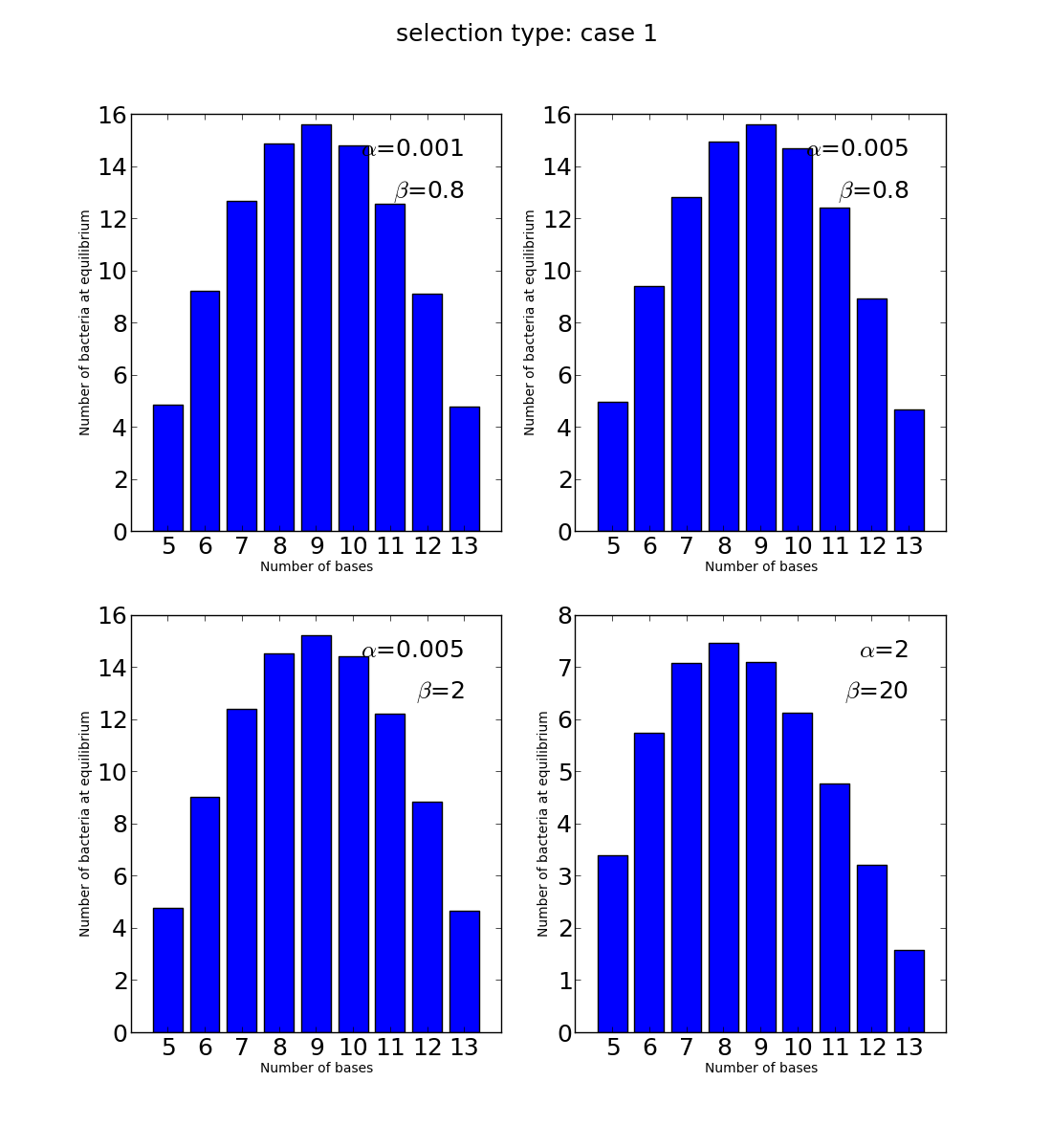

Supplement: Figure S4 — Mathematical modelling of different scenarios of selection. Allelic steady-state distributions shown for different values of death rate/mortality ds and dl as depicted in the first slide: without selection (case 1), selection ON (case 2) and OFF (case 3), tract length dependent death rate (case 4), and combinations of ON/OFF selection and tract length dependent death rate (case 5 and 6). Panels A–D in each case 1–6 illustrate the allelic steady-state distributions of tract lengths following comparable values of tract length dependent instability (α) and tract length independent instability (β). Where K = 100 and μ = 5. (ZIP) [file pone.0101637.s004.zip › FigureS4_case1.tiff]

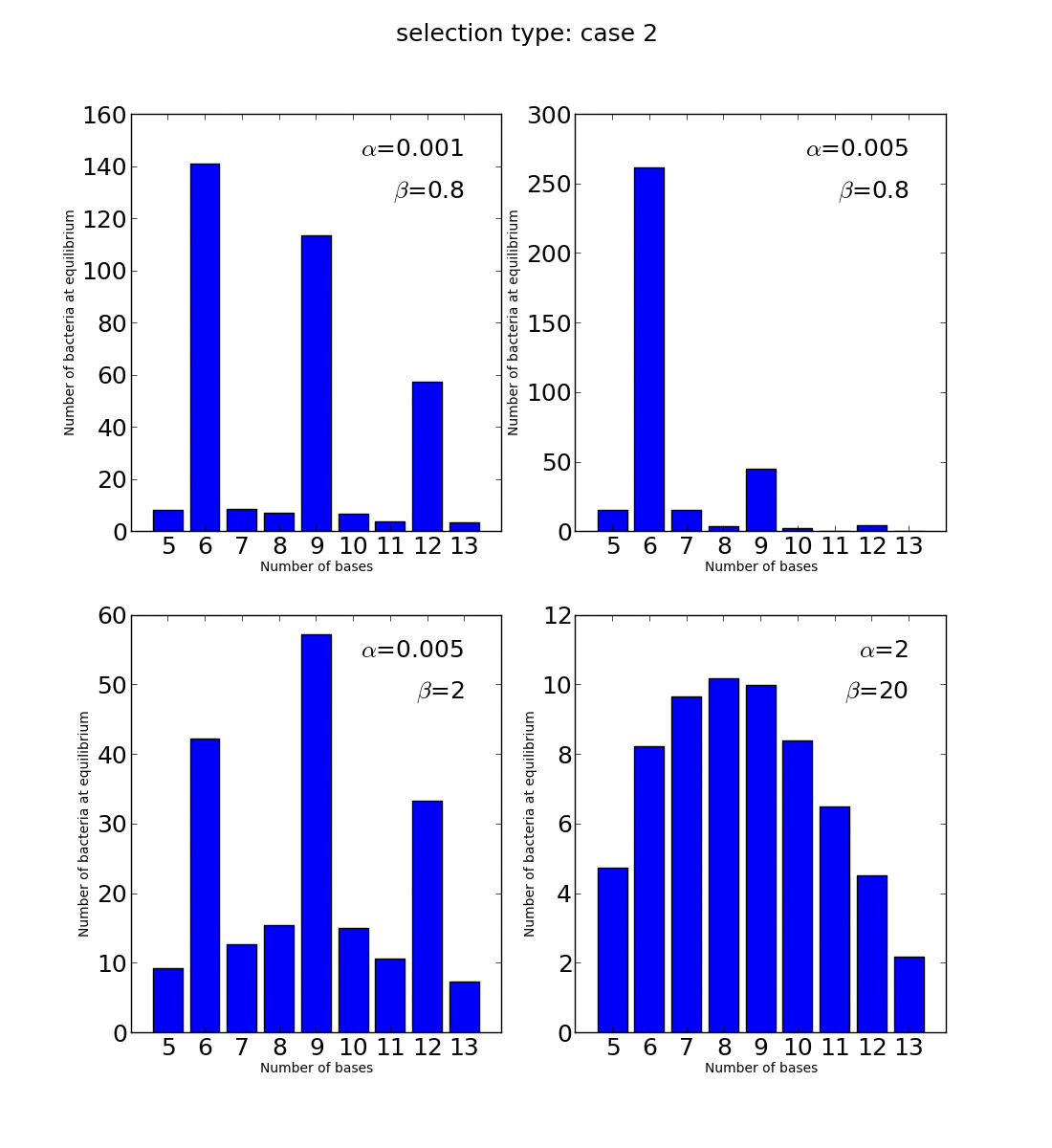

Supplement: Figure S4 — Mathematical modelling of different scenarios of selection. Allelic steady-state distributions shown for different values of death rate/mortality ds and dl as depicted in the first slide: without selection (case 1), selection ON (case 2) and OFF (case 3), tract length dependent death rate (case 4), and combinations of ON/OFF selection and tract length dependent death rate (case 5 and 6). Panels A–D in each case 1–6 illustrate the allelic steady-state distributions of tract lengths following comparable values of tract length dependent instability (α) and tract length independent instability (β). Where K = 100 and μ = 5. (ZIP) [file pone.0101637.s004.zip › FigureS4_case2.tif]

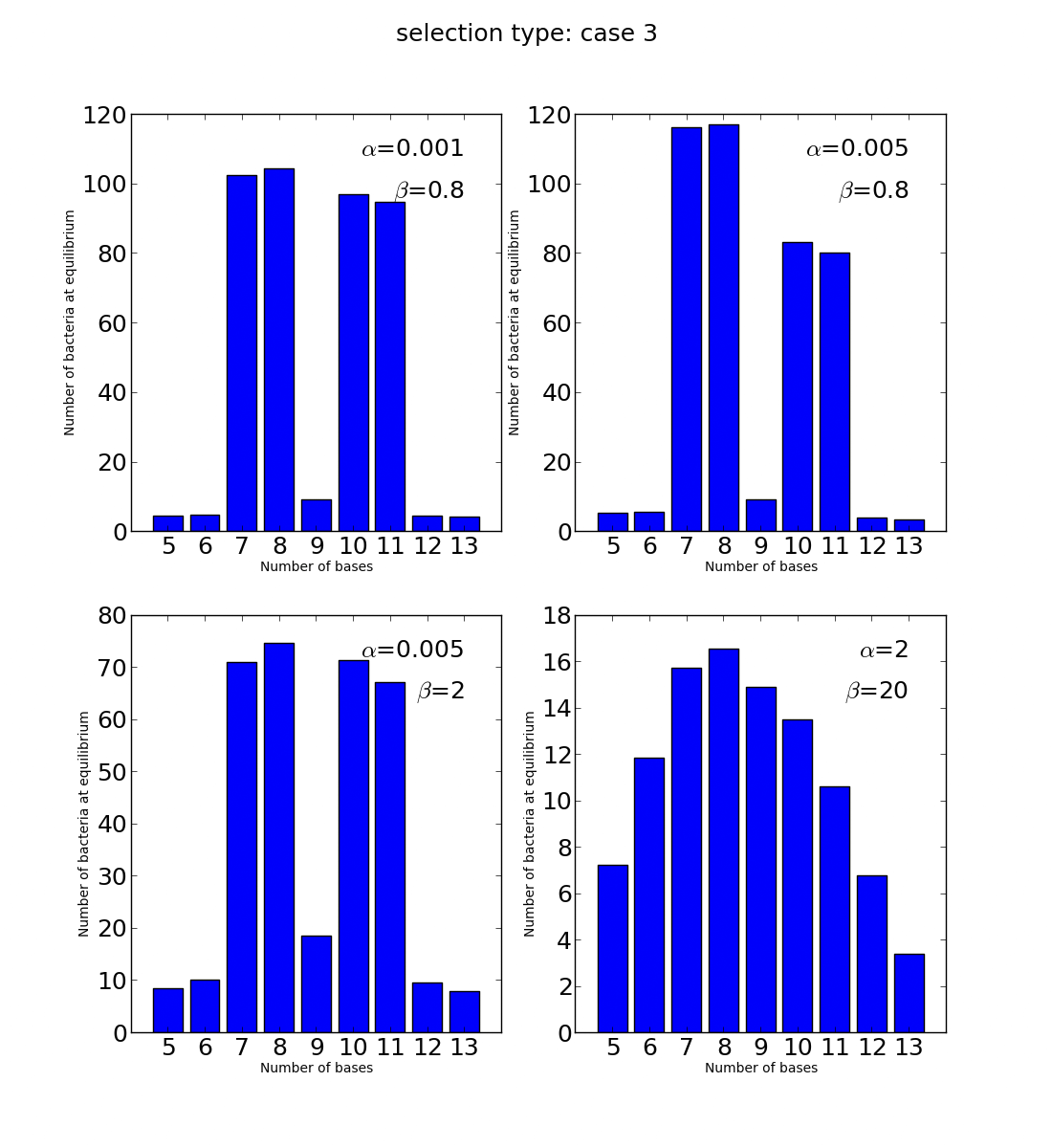

Supplement: Figure S4 — Mathematical modelling of different scenarios of selection. Allelic steady-state distributions shown for different values of death rate/mortality ds and dl as depicted in the first slide: without selection (case 1), selection ON (case 2) and OFF (case 3), tract length dependent death rate (case 4), and combinations of ON/OFF selection and tract length dependent death rate (case 5 and 6). Panels A–D in each case 1–6 illustrate the allelic steady-state distributions of tract lengths following comparable values of tract length dependent instability (α) and tract length independent instability (β). Where K = 100 and μ = 5. (ZIP) [file pone.0101637.s004.zip › FigureS4_case3.tif]

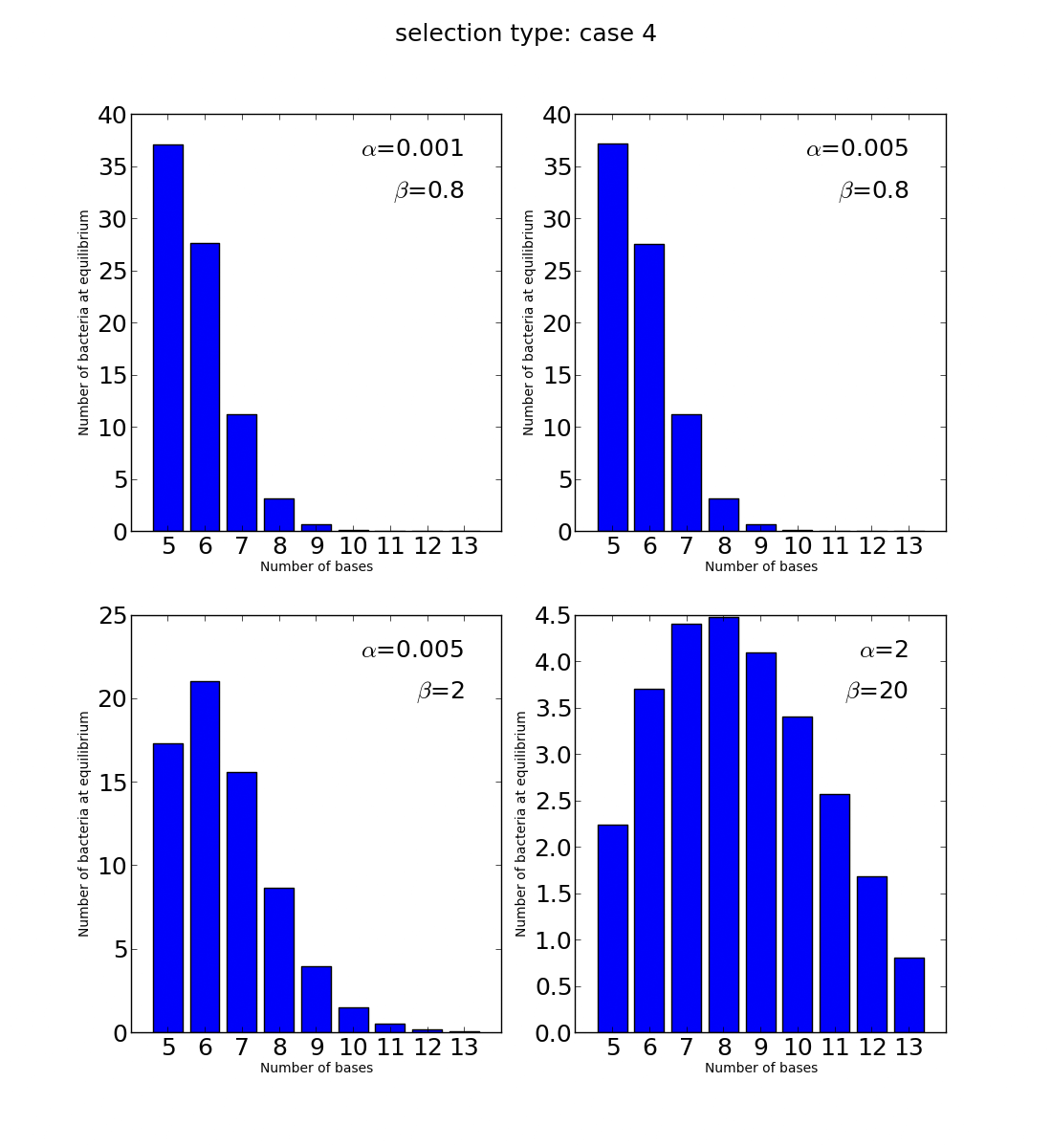

Supplement: Figure S4 — Mathematical modelling of different scenarios of selection. Allelic steady-state distributions shown for different values of death rate/mortality ds and dl as depicted in the first slide: without selection (case 1), selection ON (case 2) and OFF (case 3), tract length dependent death rate (case 4), and combinations of ON/OFF selection and tract length dependent death rate (case 5 and 6). Panels A–D in each case 1–6 illustrate the allelic steady-state distributions of tract lengths following comparable values of tract length dependent instability (α) and tract length independent instability (β). Where K = 100 and μ = 5. (ZIP) [file pone.0101637.s004.zip › FigureS4_case4.tif]

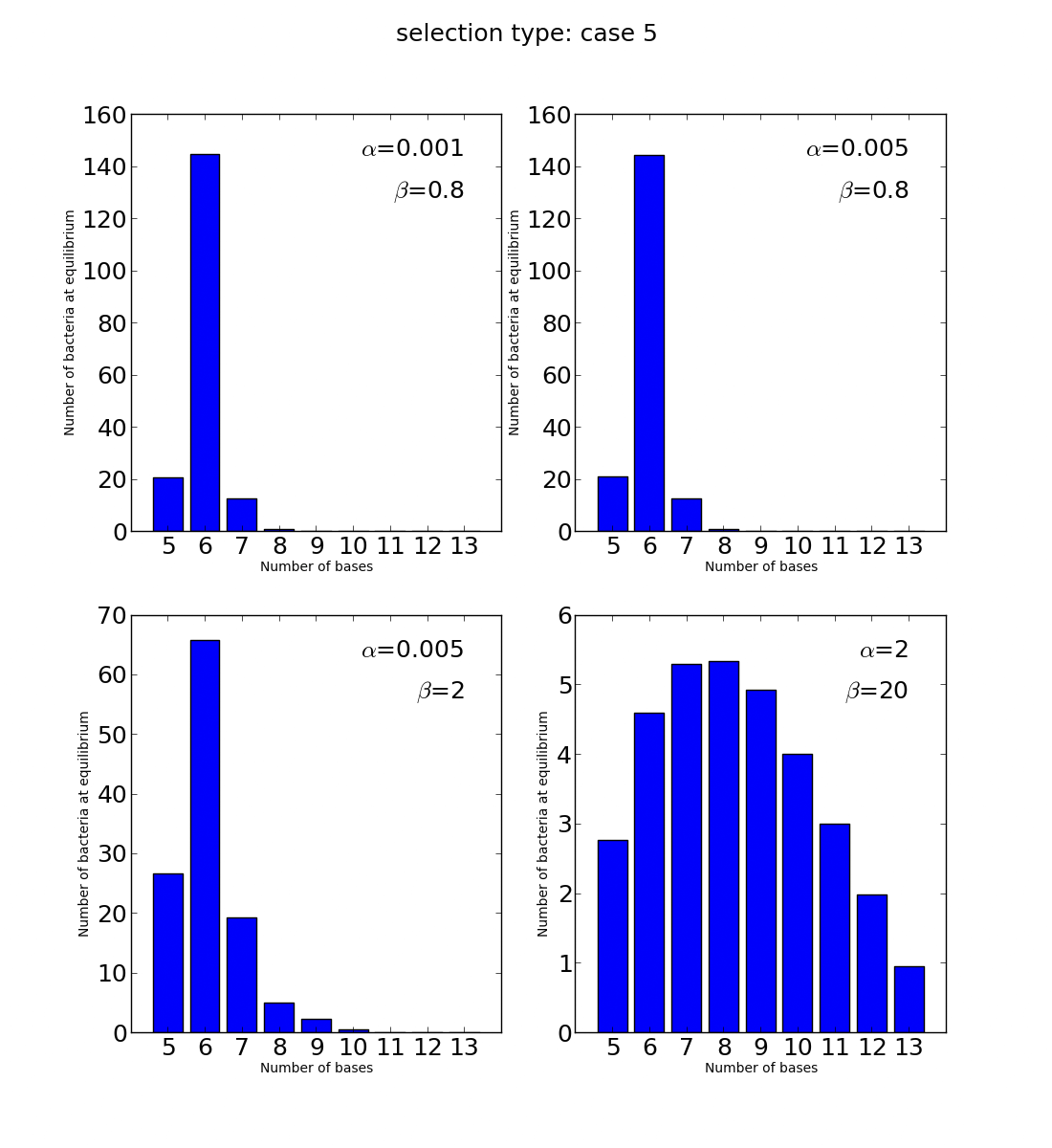

Supplement: Figure S4 — Mathematical modelling of different scenarios of selection. Allelic steady-state distributions shown for different values of death rate/mortality ds and dl as depicted in the first slide: without selection (case 1), selection ON (case 2) and OFF (case 3), tract length dependent death rate (case 4), and combinations of ON/OFF selection and tract length dependent death rate (case 5 and 6). Panels A–D in each case 1–6 illustrate the allelic steady-state distributions of tract lengths following comparable values of tract length dependent instability (α) and tract length independent instability (β). Where K = 100 and μ = 5. (ZIP) [file pone.0101637.s004.zip › FigureS4_case5.tif]

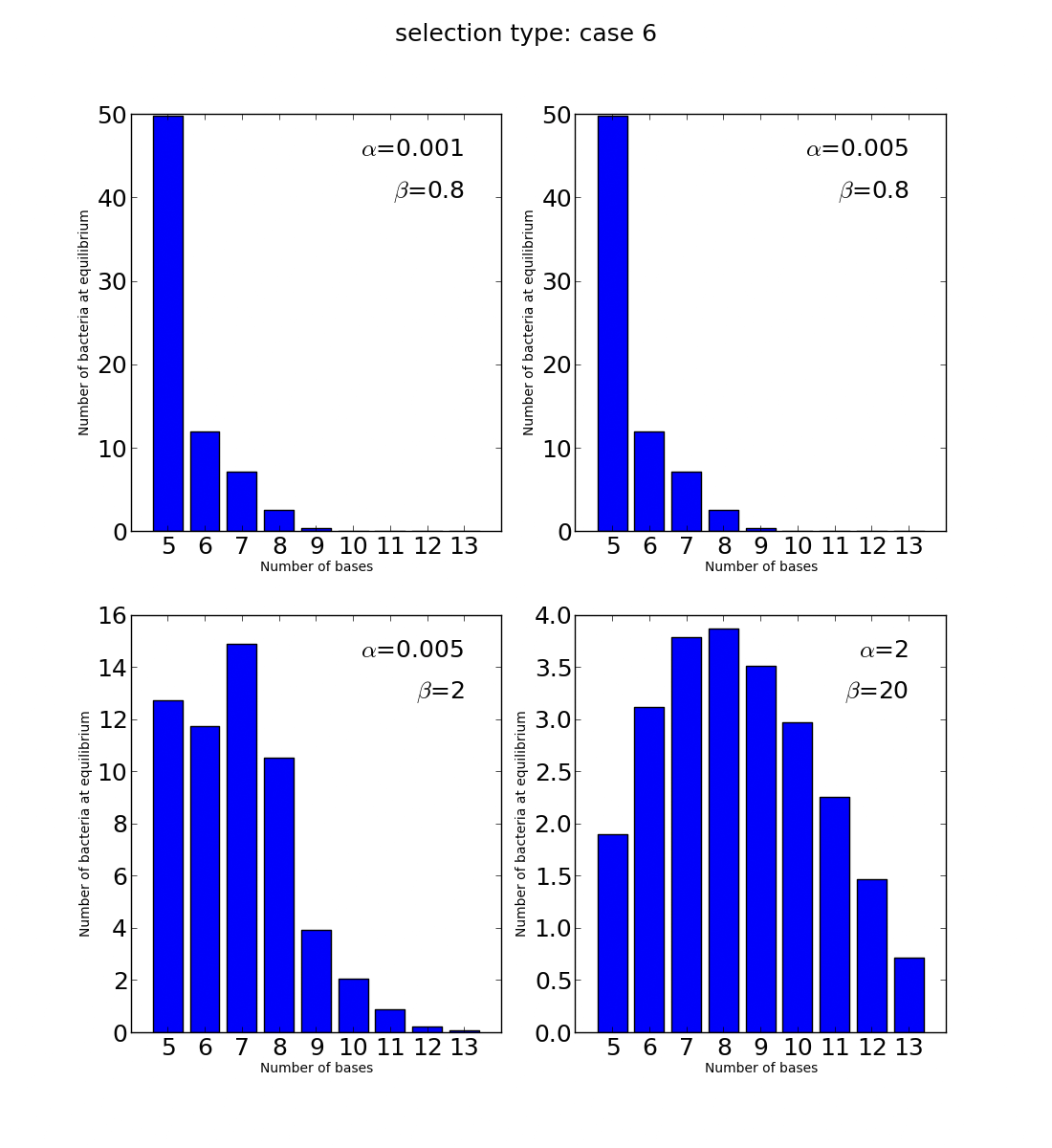

Supplement: Figure S4 — Mathematical modelling of different scenarios of selection. Allelic steady-state distributions shown for different values of death rate/mortality ds and dl as depicted in the first slide: without selection (case 1), selection ON (case 2) and OFF (case 3), tract length dependent death rate (case 4), and combinations of ON/OFF selection and tract length dependent death rate (case 5 and 6). Panels A–D in each case 1–6 illustrate the allelic steady-state distributions of tract lengths following comparable values of tract length dependent instability (α) and tract length independent instability (β). Where K = 100 and μ = 5. (ZIP) [file pone.0101637.s004.zip › FigureS4_case6.tif]

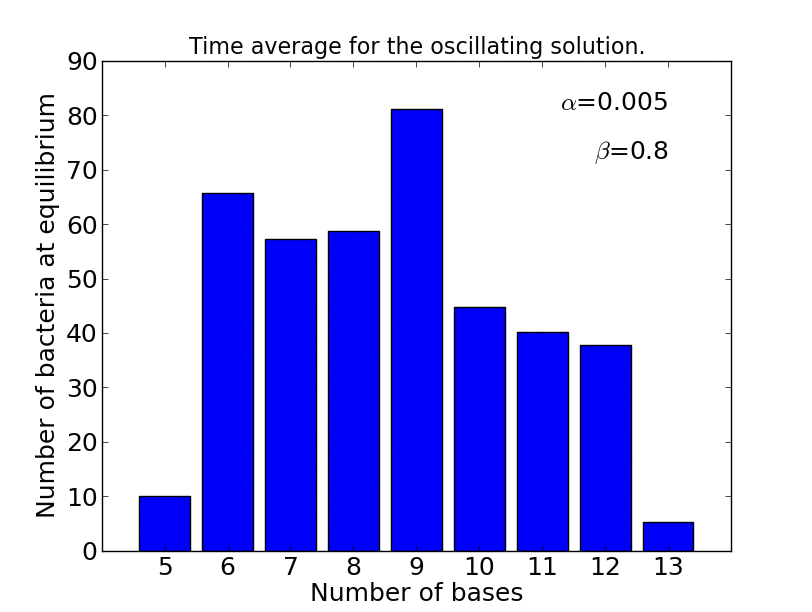

Supplement: Figure S5 — Time average of the mathematical modelled population with oscillating ON/OFF selection shown in Video S1. Allelic time average distribution of the mathematical modelled population with oscillating ON/OFF selection (as shown in Video S1). In the modelled population K = 100, μ = 5, ds = 1 and dl = 5, where ds and dl oscillates for tract lengths that are multiple of three (6, 9, 12) and not multiple of three (5, 7, 8, 10, 11 & 13). (TIF) [file pone.0101637.s005.tif]

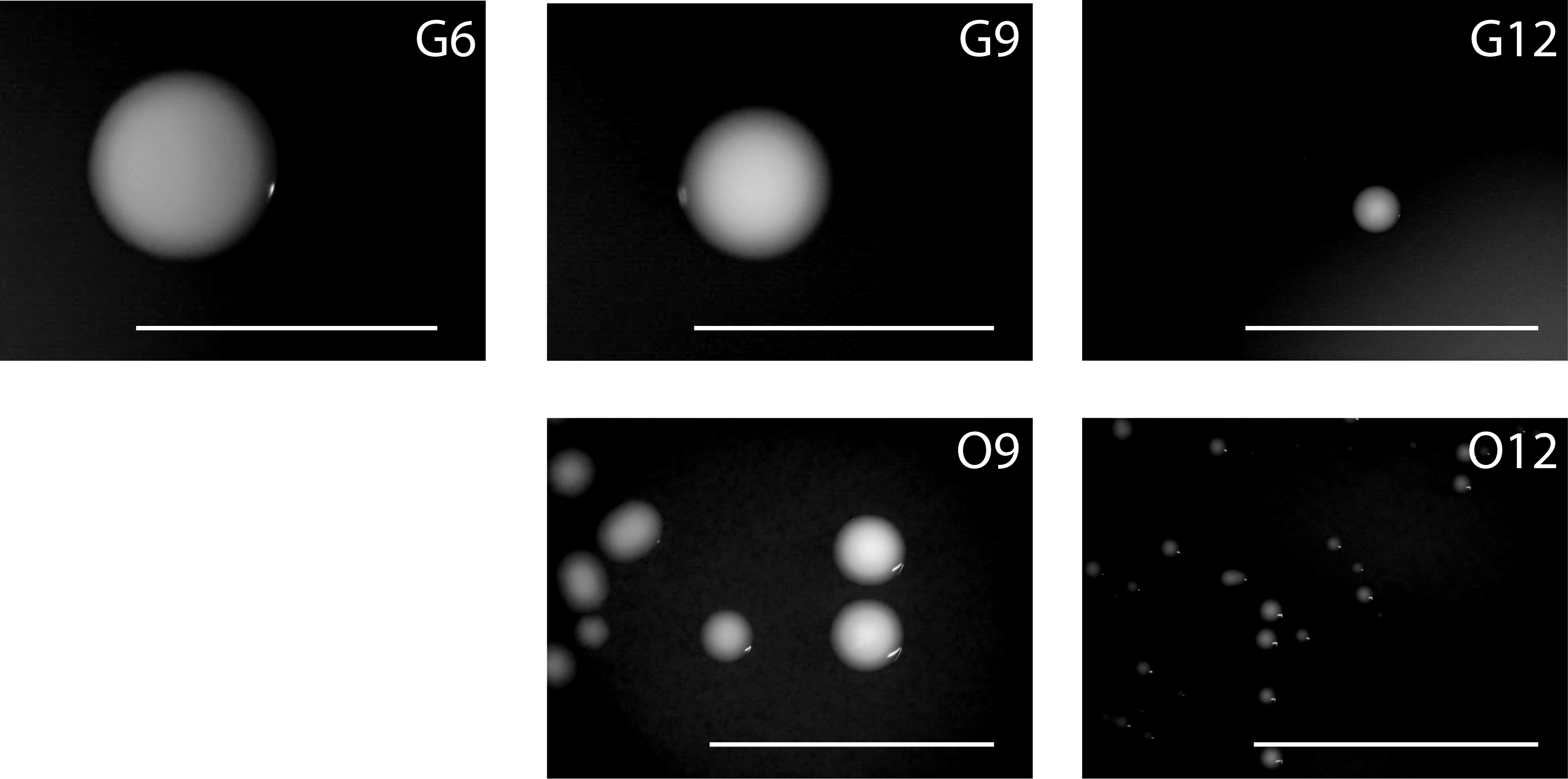

Supplement: Figure S6 — Colonies after 48 hours of growth on Blood Spc50 plates for the panel of in-frame tract lengths in wt and non-repeat controls. Depicting a selection of colonies representing the in-frame tracts G6 (for G5/G7), G9 (for G8/G10) and G12 (for G11/G13) and the non-repeat repeat tracts O9 (9 nt) and O12 (12 nt), the white bars represent 10 mm. Tract length constructs as described in Table 1 and 2. (TIF) [file pone.0101637.s006.tif]
