# Supplementary material for: Mathematical and Live Meningococcal Models for Simple Sequence Repeat Dynamics – Coherent Predictions and Observations
Source: PLoS One. 2014 Jul 7;9(7):e101637. doi: 10.1371/journal.pone.0101637 (PMC4085013; doi:10.1371/journal.pone.0101637)
Supplement: Table S1 — List of putative phase variable loci in three Mc strains. (DOC) [file pone.0101637.s007.doc]

**Table S1. List of putative phase variable loci in three Mc strains.**

| **Gene name** | **Homologous genes in strains*** | | | **Repeat‡** | **Frame‡‡** | **Repeat tract length in strains**** | | | **Reference** |
| --- | --- | --- | --- | --- | --- | --- | --- | --- | --- |
|  | **MC58** | **Z2491** | **FAM18†** |  |  | **MC58** | **Z2491** | **FAM18†** |  |
| Putative lipoprotein | NMB0032 | NMA0277 | NMC0009 | (A) | Pro | 11 | 11 | 11 | [1–4] |
| *pilC2* | NMB0049 | NMA0293 | NMC0033 | (G) | Out | 11 | 11 | 13 | [1,3,5,6] |
| Hypothetical protein | NMB0065 | - | - | (T) | Pro | 10 | NH | NH | [1–4,7] |
| *siaD* | NMB0067 | - | NMC0051 | (C) | In | 7 | NH | NR | [1,3,5,7–9] |
| *mtfB* | NMB0098 | - | NMC0086 | (G)/(A) | Out | (G)7 | NH | (A)7 | [1–4] |
| *pgtA / pglA* | NMB0218 | NMA0048 | - | (G) | In | 11 | 14 | NH | [1,3,6,7] |
| *dinG* | NMB0287 | NMA2200 | NMC0293 | (C) | In | 7 | 6 | 7 | [2] |
| Hypothetical protein | NMB0300 | NMA2186 | NMC1879 | (G) | (Out) | 6 | 6 | 7 | [1,4] |
| Hypothetical protein | NMB0368 | NMA2117 | NMC1801 | (A) | In | 11 | 8 | 8 | [1,2,4,7] |
| *dca / pptA* | NMB0415 | NMA2069 | NMC1750 | (G) | Out | 9 | 9 | 8 | [1,3,4,6,7,10,11] |
| *amiC* | NMB0456 | NMA2028 | NMC1694 | (C) | In | NR | 7 | 7 | [2,3] |
| Hypothetical protein | NMB0488 | - | NMC1424 | (C) | In | 7 | NH | NR | [1–4] |
| Hypothetical protein | NMB0593 | NMA0796 | NMC0535 | (C) | In | 7 | 7 | 7 | [1–4] |
| *potD-2* | NMB0623 | NMA0831 | NMC0567 | (C) | In | NR | 7 | NR | [2,3] |
| *hsdS* | NMB0831 | NMA1040 | - | (G) | Out | 7 | 8 | NH | [1–6] |
| Hypothetical protein | NMB0970 | NMA1167 | - | (G) | Out | 7 | 7 | NH | [1,3,4] |
| *pntA* | NMB0980 | NMA1177 | NMC0960 | (C) | Pro | NR | NR | 12 | [2] |
| Type II restriction enzyme | NMB1032 | NMA1454 | - | (C) | In | 6 | NR | NH | [1,4] |
| Hypothetical protein | NMB1033 | NMA1453 | NMC1180 | (A) | In | 7 | 9 | 9 | [2] |
| *opc* | NMB1053 | NMA1251 | NMC1877 | (C) | Pro | 12 | 17 | NR | [1,3,5,6,12] |
| *net / ner* | NMB1080 | - | - | (A) | Out | 7 | NH | NH | [1–4] |
| *mesJ* | NMB1140 | - | NMC1080 | (A) | In | 8 | NH | 8 | [1,4,7] |
| *hsdM* | NMB1223 | NMA1385/  NMA1038 | NMC0295 | (A) | In | 9 | 9 | 8 | [1,3–5,7] |
| Glycosylation protein/  putative glycosyl transferase | NMB1255 | NMA1427 | NMC1156 | (G) | Out | 8 | 9 | 9 | [2,3,6] |
| Hypothetical protein | NMB1327 | NMA1541 | NMC1265 | (C) | Out | NR | NR | 6 | [2,4] |
| *rifS /nifS* | NMB1379 | NMA1594 | NMC1315 | (C) | In | 8 | NR | NR | [1,2,4] |
| *porA* | NMB1429 | NMA1642 | NMC1364 | (G) | Pro | 11 | 12 | 7 | [1,3,5,6,13] |
| *dnaX* | NMB1443 | NMA1656 | NMC1379 | (C)/(A) | In | NR | (C)7 | (A) 7 | [3] |
| Hypothetical protein | NMB1489 | - | NMC1424 | (C) | In | 7 | NH | NR | [1–4] |
| *lbpA* | NMB1540 | NMA1739 | NMC1468 | (G) | In | 8 | NR | NR | [1,2,4,5,7] |
| *hmbR* | NMB1668 | NMA1925 | NMC1586 | (G) | In/Out | 9 | 10 | 10 | [1,3,5–7,14–17] |
| Hypothetical protein | NMB1760 | - | - | (C) | (Out) | 6 | NH | NH | [1–4] |
| Hypothetical protein | NMB1786 | NMA0678/  NMA0679 | NMC0436/  NMC0891 | (A) | Pro | 9 | 8 | 8 | [1–4] |
| *pglB2* | NMB1820 | NMA0639 | NMC0399 | (A) | In | 6 | 6 | 8 | [18] |
| *wbpC / pglI* | NMB1836 | NMA0619 | NMC0380 | (G) | In | 13 | 10 | 15 | [1–4,6] |
| *pilC1* | NMB1847 | NMA0609 | NMC0371 | (G) | In/Out | 14 | 13 | 10 | [1,3,5,6] |
| Hypothetical protein | NMB1877 | NMA0579 | NMC0342 | (G) | In | NR | 7 | NR | [2,3] |
| *lgtA* | NMB1929 | NMA0524 | NMC1902 | (G) | In | 14 | NR | NR | [1,5] |
| Hypothetical protein | NMB1931 | NMA0522 | - | (G) | (Out) | 7 | NR | NH | [1,4,5] |
| *spr / aspA* | NMB1969 | NMA0478 | NMC1943 | (C) | In | 10 | 10 | 9 | [1–4,6,7,9] |
| *fetA / frpB* | NMB1988 | NMA0453 | NMC1963 | (C) | Pro | 11 | NR | 6 | [1,4,5,7] |
| *sprA* | NMB1998 | - | NMC0651 | (C) | Out | 9 | NH | NR | [1,3–5,7] |
| Hypothetical protein | NMB2008 | - | - | (C) | Pro | 7 | NH | NH | [1–4,7] |
| *lgtG* | NMB2032 | - | NMC2011 | (C) | Out | 12 | NH | 14 | [1,3,5,7,9,19] |
| Putative phosphatase | NMB2033 | NMA0405 | NMC2014 | (G) | In/Out | NR | 12 | 12 | **†** |
| Hypothetical protein | - | NMA0640 | NMC0400 | (C) | In | NH | 7 | NR | [3] |
| *pglG* | - | NMA0641 | NMC0401 | (C) | Out | NH | 14 | 22 | [3,6] |
| *hpuAB* | - | NMA0475/  NMA0474 | NMC1946/  NMC1947 | (G) | In | NH | 11 | 10 | [3,5,6,14,15,17] |
| Putative acetyltransferase | - | NMA0407 | NMC2013 | (G) | Out | NH | 12 | 12 | [2,3,6] |
| Hypothetical protein | - | NMA0132 | - | (C) | Pro | NH | 9 | NH | [2,3,6] |
| Hypothetical protein | - | NMA1436 | NMC1161 | (C)/(T)/(AC) | In | NH | 7/6/4 | 6/6/4 | [2,3] |
| *omp85* | NMB0182 | NMA0085 | NMC0173 | (AC) | In | 4 | 4 | 4 | [1,3,4,7] |
| *vapA* | NMB0312 | NMA2175 | NMC1859 | (AAGC) | Out | 9 | 8 | 32 | [1–3,6] |
| Hypothetical protein | NMB0432 | NMA2053 | - | (TA) | In | 4 | NR | NH | [1,3,4,7] |
| *opaA* | NMB0442 | - | - | (CTTCT) | Out | 10 | NH | NH | [1,3,5] |
| Hypothetical protein | NMB0471 | NMA2014 | NMC1677 | (AC) | In | 4 | 4 | 4 | [1,3,4] |
| Hypothetical protein | NMB0486 | NMA1999 | NMC1661 | (C)(N)(G) | Out | (C)6(N)10(G)7 | (C)6(N)10(G)7 | (C)6(N)10(G)7 | [1,3,4] |
| *pglE* | NMB0624 | NMA0832 | NMC0568 | (CAACAAA) | In | 34 | 26 | 28 | [1,3,4,6,18] |
| Type II restriction enzyme | NMB0726 | - | - | (TG) | In | 4 | NH | NH | [1,3,4] |
| *opa* | NMB0926 | NMA1676 | NMC0903 | (CTTCT) | Out | 17 | 7 | 10 | [1,3,5,6] |
| *funZ* | NMB0961 | - | - | (CAAAT) | In | 5 | NH | NH | [1,3,4,7] |
| *mod* | NMB1261 | NMA1467 | NMC1194 | (CCCAA) | Out | 16 | 8 | 15 | [1,3–6] |
| Hypothetical protein | NMB1265 | - | - | (GAAA) | In | 3 | NH | NH | [1,3] |
| Hypothetical protein | NMB1275 | NMA1480 | NMC1205 | (AGCA) | (Out) | 3 | 3 | 3 | [1,2,4] |
| *tra* | NMB1277 | NMA1483 | NMC1208 | (TG) | In | 4 | 4 | 4 | [1,3,4,7] |
| *mod* | NMB1375 | NMA1589 | NMC1310 | (CAGC) | Out | 20 | 3 | 25 | [1,4,5] |
| *frpC* | NMB1407 | - | - | (ATAACAAA) | In | 4 | NH | NH | [1–4] |
| *opaA* | NMB1465 | NMA1890/  NMA2043 | NMC1551/  NMC1403 | (CTTCT) | Out | 14 | 9/11 | 7/12 | [1,3,5,6] |
| Hypothetical protein | NMB1507 | NMA1707 | NMC1436 | (CAAG) | In/Out | 11 | NR | NR | [1–3] |
| *virG* | NMB1525 | NMA1725 | NMC1454 | (AAGC) | Out | 5 | 4 | 7 | [1,2,7] |
| Hypothetical protein | NMB1543/  NMB1634 | NMA1792/  NMA0782 | NMC1861/  NMC1709/  NMC0277/  NMC0031 | (C)(N)(G) | Pro | (C)8(N)9/10 (G)7 | (C)10 (N)10(G)7 | (C)9(N)9G)7 | [1,3,4,6] |
| *opa* | NMB1636 | NMA1888 | NMC1719 | (CTTCT) | Out | 11 | NR | 12 | [1,3,5] |
| *fixP* | NMB1723 | NMA1977 | - | (AT) | In | 5 | 5 | NH | [1,3,4,7] |
| Glutaredoxin 2 | NMB1734 | NMA1990 | NMC1654 | (TGCG) | In | 3 | 3 | 2 | [1,4] |
| Hypothetical protein | NMB1741 | - | - | (C)(N)(G) | Out | (C)6(N)10(G)7 | NH | NH | [1,3,4] |
| Hypothetical protein | NMB1893 | NMA0585 | NMC0348 | (TTCC) | Out | 4 | 4 | NR | [1,2,4,7] |
| *plsX* | NMB1913 | NMA0542 | NMC0310 | (TTCC) | In | 3 | 3 | NR | [1,2,4] |
| *aipI* | NMB1985 | NMA0457 | NMC1959 | (GGCA) | In | 3 | NR | 3 | [1,4,7] |
| *nadA* | NMB1994 | - | NMC1969 | (TAAA) | Pro | 9 | NH | 9 | [1,3,4,7,9,20] |

† This study

* Genes in MC58, Z2491 and FAM18, homologous genes are grouped when appropriate, - = No homologue in strain.

**‡** Repeated motif, nucleotide or sequence, / = Different motifs identified, N = Non-repeated nucleotide sequence.

**‡‡**Location of the repeat, Pro = Promoter region, In = In frame intragenic region, Out = Out of frame intragenic region, (Out) = Out of frame intragenic region in the most probable open reading frame, In/Out = Not determined reading frame.

** Tract length of the putative phase variable tract, NH = No homologue in strain, NR = No repeat in homologue sequence, homopolymeric repeats ≤7 for all three strains were excluded if the same repeat length was observed in homologous genes in other available strains (*N. meningitidis* alpha14, *N. meningitidis* 053442, *N. gonorrhoeae* FA 1090 & *N. gonorrhoeae* NCCP11945).

References:

1. Saunders NJ, Jeffries AC, Peden JF, Hood DW, Tettelin H, et al. (2000) Repeat-associated phase variable genes in the complete genome sequence of Neisseria meningitidis strain MC58. Mol Microbiol 37: 207–215.

2. Bentley SD, Vernikos GS, Snyder LAS, Churcher C, Arrowsmith C, et al. (2007) Meningococcal genetic variation mechanisms viewed through comparative analysis of serogroup C strain FAM18. PLoS Genet 3: e23.

3. Snyder LAS, Butcher SA, Saunders NJ (2001) Comparative whole-genome analyses reveal over 100 putative phase-variable genes in the pathogenic Neisseria spp. Microbiology 147: 2321–2332.

4. Tettelin H, Saunders N, Heidelberg J (2000) Complete genome sequence of Neisseria meningitidis serogroup B strain MC58. Science (80- ) 1809. doi:10.1126/science.287.5459.1809.

5. Bayliss CD, Field D, Moxon ER (2001) The simple sequence contingency loci of Haemophilus influenzae and Neisseria meningitidis. J Clin Invest 107: 657–666.

6. Parkhill J, Achtman M, James KD, Bentley SD, Churcher C, et al. (2000) Complete DNA sequence of a serogroup A strain of Neisseria meningitidis Z2491. Nature 404: 502–506. doi:10.1038/35006655.

7. Martin P, Van De Ven T, Mouchel N, Jeffries AC, Hood DW, et al. (2003) Experimentally revised repertoire of putative contingency loci in Neisseria meningitidis strain MC58: evidence for a novel mechanism of phase variation. Mol Microbiol 50: 245–257.

8. Hammerschmidt S, Müller A, Sillmann H, Miihlenhoff M, Borrow R, et al. (1996) Capsule phase variation in Neisseria meningitidis serogroup B by slipped-strand mispairing in the polysialyltransferase gene (siaD): correlation with bacterial invasion and the outbreak of meningococcal disease. Mol Microbiol 20: 1211–1220.

9. Martin P, Sun L, Hood DW, Moxon ER (2004) Involvement of genes of genome maintenance in the regulation of phase variation frequencies in Neisseria meningitidis. Microbiology 150: 3001–3012. doi:10.1099/mic.0.27182-0.

10. Snyder LAS, Saunders NJ, Shafer WM (2001) A putatively phase variable gene (dca) required for natural competence in Neisseria gonorrhoeae but not Neisseria meningitidis is located within the division cell wall (dcw) gene cluster. J Bacteriol 183: 1233–1241.

11. Warren MJ, Jennings MP (2003) Identification and characterization of pptA: a gene involved in the phase-variable expression of phosphorylcholine on pili of Neisseria meningitidis. Infect Immun 71: 6892–6898.

12. Sarkari J, Pandit N, Moxon ER, Achtman M (1994) Variable expression of the Opc outer membrane protein in Neisseria meningitidis is caused by size variation of a promoter containing poly-cytidine. Mol Microbiol 13: 207–217.

13. Van der Ende A, Hopman CT, Zaat S, Essink BB, Berkhout B, et al. (1995) Variable expression of class 1 outer membrane protein in Neisseria meningitidis is caused by variation in the spacing between the-10 and-35 regions of the promoter. J Bacteriol 177: 2475–2480.

14. Richardson AR, Stojiljkovic I (2001) Mismatch repair and the regulation of phase variation in Neisseria meningitidis. Mol Microbiol 40: 645–655.

15. Richardson AAR, Stojiljkovic I (1999) HmbR, a hemoglobin-binding outer membrane protein of Neisseria meningitidis, undergoes phase variation. J Bacteriol 181: 2067–2074.

16. Alexander HL, Richardson AR, Stojiljkovic I (2004) Natural transformation and phase variation modulation in Neisseria meningitidis. Mol Microbiol 52: 771–783.

17. Alexander HL, Rasmussen AW, Stojiljkovic I (2004) Identification of Neisseria meningitidis genetic loci involved in the modulation of phase variation frequencies. Infect Immun 72: 6743–6747.

18. Power PM, Roddam LF, Rutter K, Fitzpatrick SZ, Srikhanta YN, et al. (2003) Genetic characterization of pilin glycosylation and phase variation in Neisseria meningitidis. Mol Microbiol 49: 833–847.

19. Bayliss CD, Hoe JC, Makepeace K, Martin P, Hood DW, et al. (2008) Neisseria meningitidis escape from the bactericidal activity of a monoclonal antibody is mediated by phase variation of lgtG and enhanced by a mutator phenotype. Infect Immun 76: 5038–5048.

20. Martin P, Makepeace K, Hill SA, Hood DW, Moxon ER (2005) Microsatellite instability regulates transcription factor binding and gene expression. Proc Natl Acad Sci U S A 102: 3800–3804.
